# 3D tumor spheroid microarray for high-throughput, high-content natural killer cell-mediated cytotoxicity

**DOI:** 10.1038/s42003-021-02417-2

**Published:** 2021-07-21

**Authors:** Sneha Gopal, Seok-Joon Kwon, Bosung Ku, Dong Woo Lee, Jungeun Kim, Jonathan S. Dordick

**Affiliations:** 1 Department of Chemical and Biological Engineering, and Center for Biotechnology & Interdisciplinary Studies, Rensselaer Polytechnic Institute, Troy, NY USA; 2 MBD (Medical & Bio Decision) Co., Ltd, Suwon-si, Gyeonggi-do Republic of Korea; 3 Department of Biomedical Engineering, Konyang University, Daejeon, Republic of Korea; 4 Departments of Biomedical Engineering and Biological Sciences, Rensselaer Polytechnic Institute, Troy, NY USA

**Keywords:** Tumour immunology, High-throughput screening

## Abstract

Immunotherapy has emerged as a promising approach to treating several forms of cancer. Use of immune cells, such as natural killer (NK) cells, along with small molecule drugs and antibodies through antibody dependent cell-mediated cytotoxicity (ADCC) has been investigated as a potential combination therapy for some difficult to treat solid tumors. Nevertheless, there remains a need to develop tools that support co-culture of target cancer cells and effector immune cells in a contextually relevant three-dimensional (3D) environment to provide a rapid means to screen for and optimize ADCC-drug combinations. To that end, here we have developed a high throughput 330 micropillar-microwell sandwich platform that enables 3D co-culture of NK92-CD16 cells with pancreatic (MiaPaCa-2) and breast cancer cell lines (MCF-7 and MDA-MB-231). The platform successfully mimicked hypoxic conditions found in a tumor microenvironment and was used to demonstrate NK-cell mediated cell cytotoxicity in combination with two monoclonal antibodies; Trastuzumab and Atezolizumab. The platform was also used to show dose response behavior of target cancer cells with reduced EC_50_ values for paclitaxel (an anti-cancer chemotherapeutic) when treated with both NK cells and antibody. Such a platform may be used to develop more personalized cancer therapies using patient-derived cancer cells.

## Abstract (web summary)

Gopal et al. present a high-throughput platform for analysing the cytotoxicity of various combination therapies against established cancer cell lines, focusing especially on NK cell-mediated cytotoxicity. The presented method is reported to successfully mimic the tumour microenvironment and demonstrates NK cell-mediated cell cytotoxicity using small molecule chemotherapeutic drugs in combination with monoclonal antibodies.

## Introduction

Cancer immunotherapy has emerged as an effective regimen alone or in combination with other treatments, such as surgery, chemotherapy, and radiation therapy^1^. With the rapid increase in our understanding of the immune system, an increasing number of small molecules^2^, peptides^3^, recombinant antibodies^4^, vaccines^5^, and cellular therapeutics^6^ have been applied to manipulate the immune response for cancer treatment. These immunotherapies have provided benefits in the fight against cancer, especially the application of immune checkpoint inhibitors^7^ and cell-based therapies^8^. Unfortunately, solid tumors, such as pancreatic or breast cancer, are often resistant to immunotherapy^9,10^. A major reason for this is the immunosuppressive effect of the tumor microenvironment (TME) that consists of abundant stroma, protumor macrophages, and regulatory T cells that prevent the proliferation of other effector T cells^11,12^. Together with the tumor cells, this complex ecosystem can lead to immune escape and immunotherapy resistance.

Cancer immunotherapy alone or in combination with antibodies and small molecule chemotherapeutics can, in principle, overcome immune-driven immunosuppression, with combination treatment being currently regarded as a promising approach^13^. In particular, the use of natural killer (NK) cells has been investigated for their potential to induce cytotoxicity in cancer cells^14^. However, given a large number of approved and candidate drugs, it is not feasible to compare all immunotherapy agents in a clinical setting. Therefore, various experimental ex vivo models have been developed to study combination treatment, including classical 2D cell culture models, tumor spheroids and organoids, and patient-derived xenografts^15–18^. Among these models, conventional 2D cell culture cannot effectively recapitulate the complexity of cellular interactions and mimic the in vivo TME. Furthermore, patient-derived xenografts suffer from limited scalability especially for screening multiple combination therapies^19^. Tumor spheroid and organoid models have been adapted for high throughput therapeutic drug screening^20,21^. Platform designs that incorporate tumor-immune cell cocultures in contextually relevant 3D microenvironments can prove useful for studying antibody-dependent cellular cytotoxicity (ADCC), leading to personalized therapy regimens for patients.

To effectively identify successful combination therapies, it is critical to evaluate the cytotoxic efficacies of effector cells, such as NK and T cells against target cancer cells. A ^51^Cr release assay has long been the most widely used method for quantification of ADCC by measuring the radioactivity of ^51^Cr released from dead cells^22^. However, the reproducibility, sensitivity, and specificity of the ^51^Cr release assay are not adequate because of the spontaneous release of ^51^Cr-labeled target cells^23^. In addition, the ^51^Cr release assay is unsafe for untrained researchers to handle because of its radioactivity^24,25^. Nonradioactive probes, such as Calcein-AM or lanthanide chelates have been used as alternatives to the ^51^Cr-release assay wherein cells are prelabeled with the dyes prior to treatment with effector cells and cytotoxicity is measured via release of the dye^26,27^. Nonetheless, these methods also show high levels of spontaneous release of the probes^28^, and target cell line-dependent labeling variability results from the activity of intracellular esterases^29^. Another method for evaluating ADCC is flow cytometry after labeling target and effector cells with different fluorescent probes and staining with cell viability dyes^30^. These methods specifically quantify target cell death or disappearance, which provide more reproducible and sensitive data^23,31^. However, flow cytometry often shows relatively high sample-to-sample variation^29^, and can require long processing times depending on the number of samples.

Several platforms for generating spheroids to study NK-mediated ADCC have been developed^32–38^. A majority of these platforms are either standard scaffold-free microwell plate cultures^38^ or microfluidic systems utilizing both scaffold-free and scaffold-based methods to enable cell culture^32–37^. While scaffold-free systems can be high-throughput, they do not incorporate an external matrix and require cells to generate their own extracellular matrix (ECM). This can be disadvantageous since carcinoma cells often coexist in an environment that contains a basement membrane matrix^39^. Hence a cancer-immune cell screening platform should include this component to better recapitulate the in vivo TME. In addition, with standard scaffold-free 3D cell culture methods, performing ADCC can be challenging. Removing effector cells, carrying out medium exchange, or washing target cells in such a system without disturbing the target spheroids (which can be aspirated during any of these steps) can be difficult. Microfluidic platforms have the advantage of a dynamic flow capability and can recapitulate a TME, yet typically do not always allow for rapid testing of a large number of conditions or samples simultaneously. Such testing is critical in more widespread screening, particularly for personalized immunotherapy.

We have developed herein a high throughput micropillar-microwell sandwich 3D cell culture platform to overcome the aforementioned limitations of current high-throughput methods. This 3D tumor spheroid microarray consists of microencapsulated cells in Matrigel, and thus, possesses a basement membrane component. The generation of 3D tumor spheroids are enabled on a micropillar surface to mimic the TME and to coculture target cancer cells and NK cells for investigation of ADCC. Rapid quantification of cytotoxicity is feasible without pre-labeling of target cells, separate labeling of effector and target cells with different fluorescent probes, or long processing times. Cell–cell and cell–ECM interactions are maintained in an in vitro system to mimic a TME. Finally, the platform enables rapid simultaneous testing of combinations of NK cell type with antibodies and small molecule therapeutics. As a result, we have identified several combinations of NK92-CD16 cells and antibodies that induce cytotoxicity in multiple metastatic cancer spheroids. We also investigated the dose-response behavior of cancer spheroids when exposed to a known chemotherapeutic drug, paclitaxel, in combination with antibody-treated NK92-CD16 cells. We believe this platform can serve as an effective high-throughput, high-content screening tool for the development of personalized immunotherapies involving both patient-derived target and immune cells.

## Results

### 2D cancer cell culture for ADCC using 384-pillar/well sandwich platform

**Fig. 1:**
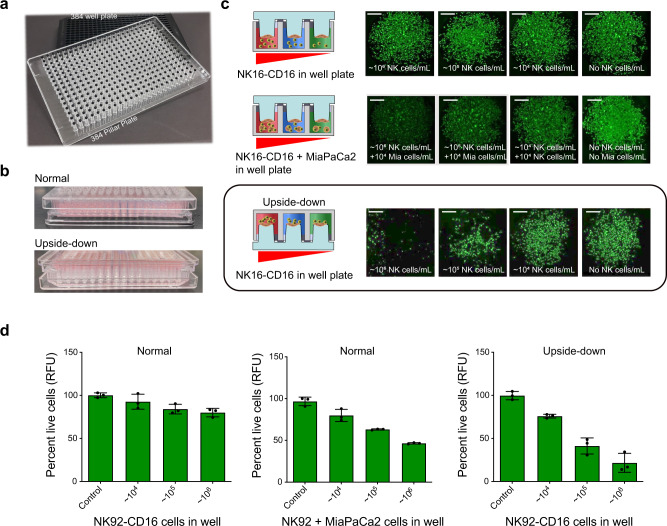
NK92-CD16 cell-mediated killing of MiPaCa2 cells using 384-pillar/well plate sandwich system. **a** Photo of 384-pillar/well plate for effector cell-mediated cytotoxicity. **b** Photo of normal and upside-down 384-pillar/well sandwich system during NK cell exposure toward 2D MiPaCa2 cell culture. Normal orientation refers to incubation of pillar surface face down on the corresponding well plate while upside-down orientation refers to pillar surface face up on the corresponding well plate. **c** NK92-CD16 cell-mediated cytotoxicity using 2D culture-based 384-pillar/well sandwich platform. The upside-down orientation provides more efficient NK cell-mediated cancer cell killing. Scale bars show 200 µm. **d** Cytotoxicity of NK92-CD16 cells against MiaPaCa-2 cells was quantified using ImageJ software after staining with Calcein-AM. The first two figures in (**d**) are in normal orientation with and without MiaPaCa-2 cells, respectively. The third figure in (**d**) is the upside-down orientation. Error bars indicate mean ± SD for *n* = 3.

ADCC of MiaPaCa-2 (pancreatic ductal adenocarcinoma cell line) was investigated initially using 2D cell culture within a 384-pillar/well sandwich platform^40^, which consists of a conventional 384-well plate along with a complementary plate with projecting pillars (Fig. 1a, b). A detailed schematic of the experimental protocol is shown in Supplementary Fig. 1. The incubation of MiaPaCa-2 cells with NK92-CD16 was performed both upside down and normally. Normal orientation refers to incubating pillar surfaces face down onto the corresponding well plate while upside-down orientation refers to incubating pillar surfaces face up onto the corresponding well plate (Fig. 1b). Direct contact between effector cells and target cells was necessary to enable the efficient killing of MiaPaCa-2 cells. Specifically, as can be seen in Fig. 1c, d, the percent of live cells were reduced to ~20% when pillars were incubated face up than with pillars face down. In addition, the presence of MiaPaCa-2 cells along with NK92-CD16 cells in the wells increased cell cytotoxicity against MiaPaCa-2 spotted on the pillars (Fig. 1b, d and Supplementary Data 1). This is likely due to granzyme release from the interaction of NK92-CD16 cells and MiaPaCa-2 cells in the well plate. Previous work has shown that direct contact between effector and target cells upregulates the expression of granzyme B^41^. In normal orientation, NK92-CD16 cells tended to settle to the bottom of the wells due to gravity. Thus, there was little direct contact between the effector and target cells. However, when the sandwiched pillar/well platform was maintained upside down, settling of NK92-CD16 cells onto the target cells on the pillar surfaces allowed for direct contact and more efficient killing. Therefore, all ADCC experiments were performed in this upside-down orientation.

**Fig. 2:**
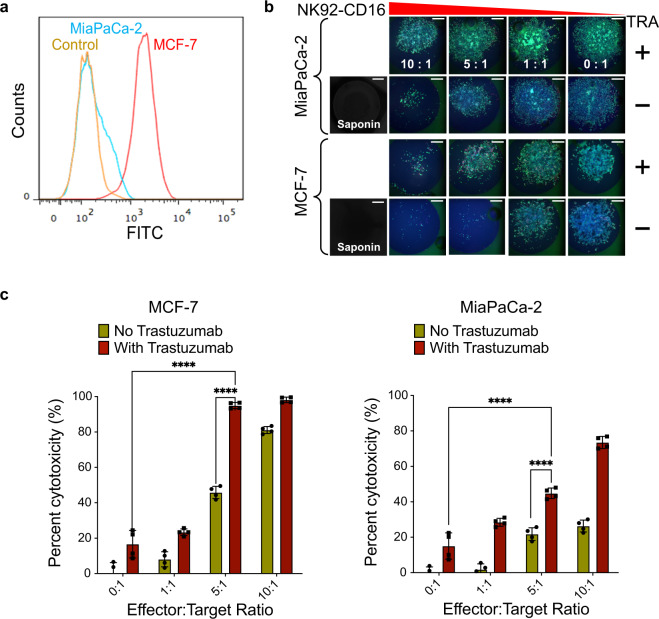
Antibody-dependent cell-mediated cytotoxicity (ADCC) using the 2D culture-based 384-pillar/well upside-down sandwich platform. **a** HER2/neu-expressing profiles of MiaPaCa-2 (cyan) and MCF-7 (red) were determined by flow cytometry after incubating with Trastuzumab-Alexa Fluor 488-conjugate, and control MCF-7 cells (orange) were incubated only with the FITC-labeled secondary antibody. **b** Images of 2D cultured target cells (MiaPaCa-2 and MCF-7 cells) stained with Calcein-AM and Hoechst 33342 on the surface of the 384-pillar plate after treating with NK92-CD16 cells and/or Trastuzumab (TRA). Scale bars show 200 µm. **c** Cytotoxicity of NK92-CD16 cells was quantified using ImageJ software via live MiaPaCa-2 (left panel) and MCF-7 (right panel) cell images stained with Calcein-AM. Percent cytotoxicity was calculated by NK cell-mediated killing/maximum killing by saponin. Error bars indicate mean ± SD for *n* = 4*.* (*p ≤ 0.05, **p ≤ 0.01, ***p ≤ 0.001, ****p ≤ 0.0001, ns not significant from unpaired student’s *t-*test and ANOVA followed by post hoc Dunnett’s test).

We then used the 2D platform to investigate the effect of NK92-CD16 cell-mediated killing of both MiaPaCa-2 and a breast cancer cell line, MCF-7, in the presence and absence of 5 µg/mL of Trastuzumab, which targets the HER2 receptor and has been used in breast cancer treatment^42^. The cells were incubated with the antibody and NK cells for 24 h. We first evaluated the expression of HER2 on both MiaPaCa-2 and MCF-7 cell lines using Alexa Fluor 488 conjugated Trastuzumab via flow cytometry (Fig. 2a), which, consistent with the literature^43,44^, indicated that MCF-7 cells expressed more HER2 than MiaPaCa-2 cells. We then evaluated the effect of ADCC using NK92-CD16 on MiaPaCa-2 and MCF-7 cells with and without Trastuzumab at three different effectors to target (E:T) ratios. Both MCF-7 and MiaPaCa-2 cells were cultured on the 384-pillar/well sandwich platform. The percent cytotoxicity was calculated using ImageJ by measuring killing using NK92-CD16 cells vs. killing using saponin. The addition of Trastuzumab along with NK92-CD16 cells enhanced killing efficiency against both cancer cell lines (Fig. 2b, c and Supplementary Data 2). Specifically, MCF-7 cells exhibited higher cytotoxicity than MiaPaCa-2 cells at the same E:T ratio in the presence of Trastuzumab with close to 90% killing at an E:T ratio of 5:1 for MCF-7 cells. This was expected given the higher expression of HER2 in MCF-7 cells than in MiaPaCa-2 cells based on flow cytometry (Fig. 2a). However, in the absence of NK92-CD16 cells, Trastuzumab caused similar cytotoxicity in both MCF-7 and MiaPaca-2 cells despite higher expression of HER2 in MCF-7 cells. This may be explained by the short-term (24 h) incubation of the antibody with the cancer cells (Fig. 2c) and measuring cell viability as opposed to proliferation. Thus, it is not surprising to observe similarly low levels of cytotoxicity with just Trastuzumab treatment alone.

These experiments show the feasibility of conducting ADCC with parallel exposure of effector cells and antibodies on the 384-pillar plate platform using conventional 2D cell culture methods. Several advantages in using this system for studying ADCC were evident, including easy removal of effector cells during the staining step as well as the absence of spontaneous release of Calcein-AM, since the cells do not require prestaining prior to treatment with NK92-CD16 cells. In addition, this 2D platform is compatible with high content imaging platforms for rapid testing of myriad therapeutic combinations. Nevertheless, since 2D platforms do not truly recapitulate the complex TME of cancer cells due to the absence of an ECM nor do they mimic hypoxic conditions, we proceeded to establish a 3D cell culture system using a sandwich pillar/well plate platform^40^.

### 3D cancer cell aggregates for NK-mediated cytotoxicity using 384-pillar/well sandwich platform

**Fig. 3:**
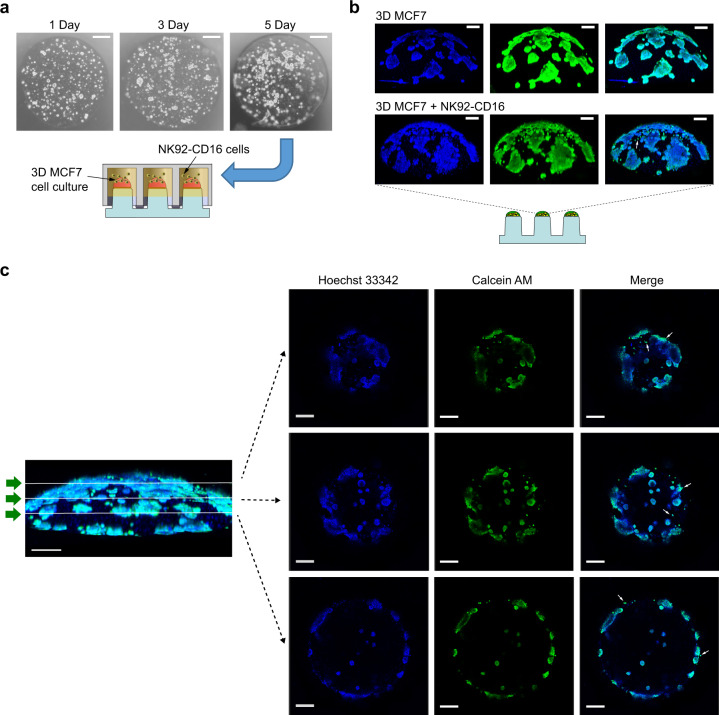
NK92-CD16 cell-mediated killing against 3D MCF-7 cell aggregates in Matrigel. **a** Microscopic image of 3D MCF-7 cell aggregates on the surface of the 384-pillar plate. NK92-CD16 cell-mediated cytotoxicity was performed using the 384-pillar/well sandwich platform. **b** Confocal microscopic images of 3D MCF-7 cell aggregates after 5 days stained with Calcein-AM and Hoechst 33342 after treating with NK92-CD16 cells (3D MCF-7 + NK92-CD16) compared to no treatment with NK92-CD16 cells (3D MCF-7). Scale bars show 200 µm. **c** Cross-sectional images after 5 days at different focal planes using Imaris software. A green arrow symbol orients the viewing direction for the cross-sections reconstructed from Z stacks. Arrows indicate possible infiltrating NK cells stained with Calcein-AM. Scale bars show 200 µm.

The pillar surfaces were first coated with dopamine hydrochloride in Tris HCl to generate a polydopamine coating. MCF-7 cells were then mixed with high concentration growth factor-reduced Matrigel (Corning Life Sciences, Corning, NY) and spotted on the coated pillar surface. The polydopamine coating on the pillar surface allows for covalent coupling between polydopamine and Matrigel proteins. The encapsulated cells grew as discrete aggregates over 5 days (Fig. 3a). At the end of 5 days, the cells were exposed to NK92-CD16 cells overnight, then stained with Calcein-AM (to label live cells) and Hoechst 33342 (to label nuclei), and imaged using confocal microscopy. A schematic of the protocol is depicted in Supplementary Fig. 2.

**Fig. 4:**
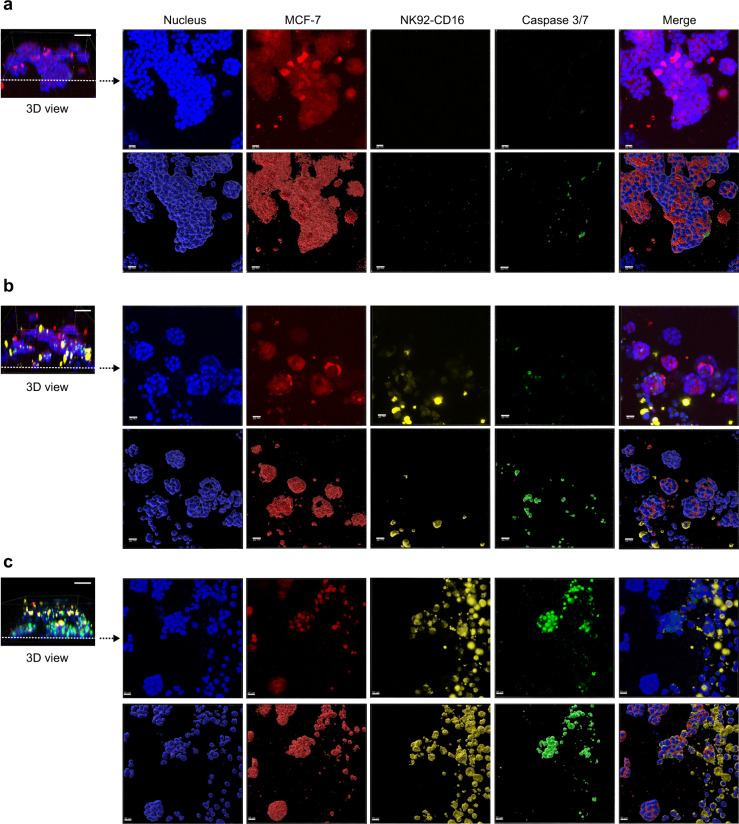
Interaction between effector cell (NK92-CD16) and 3D MCF-7 cell aggregate. Confocal microscopy images of NK cell-mediated killing against 3D MCF-7 cells entrapped in Matrigel. MCF-7 cells were pre-stained with CellTracker Orange and NK92-CD16 cells were labeled with CellTracker Deep Red. The aggregates were cultured in the presence and absence of NK92-CD16 cells. Two-time points were investigated in the presence of NK92-CD16 cells. Apoptotic cells (green) were stained with CellEvent Caspase 3/7 dye and the total cell nucleus (blue) was stained with Hoechst 33342. MCF-7 cells are shown in red and NK92-CD16 cells are shown in yellow. Both cross-sectional images (i–v) and a volume reconstructed portion of the cross-section (vi–x) are shown. **a** MCF-7 aggregates cultured in the absence of NK92-CD16 cells.; **b** MCF-7 aggregates cultured in the presence of NK92-CD16 cells for 1 h; and **c** MCF-7 aggregates cultured in the presence of NK92-CD16 cells for 24 h. Scale bars in 3D images (left panel) show 50 µm. Scale bars in top views (right panel) show 20 µm (**a** and **b**) and 30 µm (**c**), respectively.

The effect of NK92-CD16 exposure on MCF-7 aggregates in the 3D microenvironment on the pillar spot is shown in Fig. 3b, c. There was a ~60% reduction in the Calcein-AM staining for MCF-7 aggregates in the presence of NK92-CD16 cells (Supplementary Fig. 3a and Supplementary Data 3). This indicates that NK92-CD16 cells can induce cytotoxicity of MCF-7 aggregates even when the target cells are encapsulated in a 3D matrix. To better distinguish MCF-7 aggregates from NK92-CD16 cells, a four-color confocal experiment was performed. MCF-7 cells were pre-stained with CellTracker Orange prior to aggregate formation, while NK92-CD16 cells were pre-stained with CellTracker Deep Red prior to exposure to the MCF-7 aggregates. Three conditions were tested wherein MCF-7 aggregates were not exposed to NK92-CD16 cells or exposed to NK92-CD16 cells for 1 or 24 h. All cells were then stained with CellEvent Caspase 3/7 Green to label dead cells and Hoechst 33342 to label nuclei; MCF-7 aggregates are red and NK92-CD16 cells are yellow (Fig. 4a–c). Without the addition of NK92-CD16 cells, there was essentially no caspase 3/7 expression, indicating that the MCF-7 cells remained viable in 3D. However, with increasing exposure time, there was a greater percentage of cells that became apoptotic and expressing Caspase 3/7. Specifically, the percentage of apoptotic cells increased from ~10 to ~50% over 24 h (Supplementary Fig. 3b and Supplementary Data 3). Furthermore, there was a migration of the NK92-CD16 toward the MCF-7 aggregates as indicated by the colocalization of NK92-CD16 cells with MCF-7 aggregates even at 1 h (Fig. 4b). Ameboid migration of leukocytes has been previously demonstrated for the rapid movement of highly motile cell types^45^. Such a process does not rely on the use of proteases to degrade the surrounding matrix but rather reorganization of the cytoskeleton to drive movement and as a result, is more rapid than mesenchymal migration^46^. This could explain the presence of NK92-CD16 cells around the tumor aggregates within 1 h. Nevertheless, at 24 h, there was a greater concentration of NK92-CD16 cells surrounding the aggregates, indicating that they continue to migrate towards MCF-7 cells with time. These experiments established that NK92-CD16 cell-mediated cytotoxicity can be performed using the 384-pillar/well sandwich platform. However, as a next step, we investigated the formation of 3D spheroids to obtain a more contextually relevant system.

### Uniform sized 3D tumor spheroid micropillar array for NK-mediated cytotoxicity

**Fig. 5:**
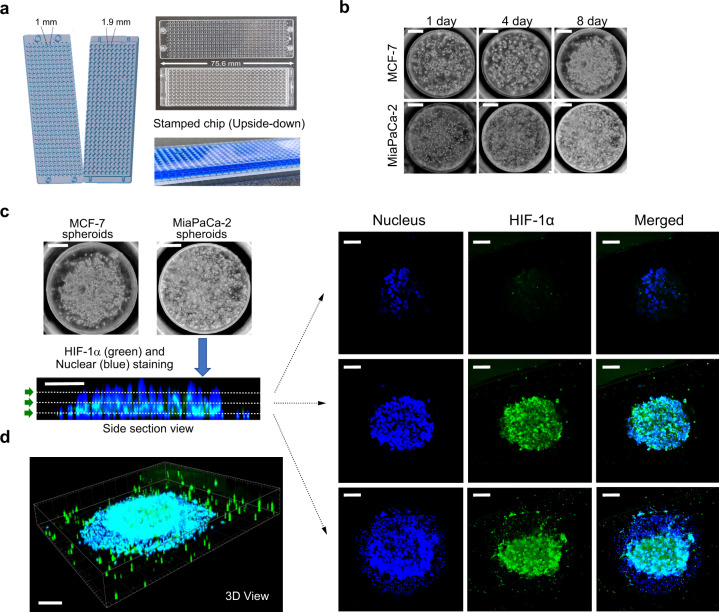
Generation of 3D tumor spheroid micropillar array. **a** Photo of 330-micropillar/well chip for effector cell-mediated cytotoxicity. The diameters of micropillar and microwell array spots are 1 and 1.9 mm, respectively. **b** Generation of 3D tumor spheroid by printing high-density cells (~2500 cells/250 nL) in Matrigel, followed by culturing cancer cells for up to 8 days. **c** Expression of HIF-1α inside MiaPaCa-2 3D tumor spheroids. Confocal microscopy images of MiaPaCa-2 3D tumor spheroids stained with Hoechst 33342 and labeled with HIF-1α antibody via immunofluorescence. Top views of cross-sectional images in a 3D tumor spheroid at different focal planes (top, middle, and bottom) using Imaris software. A green arrow symbol orients the viewing direction for the cross-sections reconstructed from Z stacks. **d** 3D view of a 3D tumor spheroid after staining the nucleus and labeling HIF1α. Scale bars show 300 µm.

We proceeded to use the 330-micropillar/microwell platform (Fig. 5a) to generate target cell spheroids. This is similar to the 384-pillar/well sandwich platform, albeit smaller in pillar surface area and overall size of the platform. The 330-micropillar and 330-microwell chips are approximately the size of a glass slide and the diameter of each pillar and well is 1 and 1.9 mm, respectively. Due to the reduced surface area, smaller volumes (250 nL) of Matrigel-cell mixtures at a high cell concentration (1 × 10^7^ cells/mL) were spotted on the surface of the micropillars. Multiple concentrations were tested ranging from 2.5 × 10^6^ cells/mL to 1 × 10^7^ cells/mL. We chose 1 × 10^7^ cells/mL because it gave us the best spheroids in terms of morphology, uniformity, and cell staining. We hypothesized that at high cell densities, as the target cells divide on the chip, multiple mini aggregates can combine to form a larger spheroid. The micropillar chips were incubated at 37 °C in a humidity chamber for 15 min following cell spotting to cause gelation of the spots and were then stamped with the corresponding microwell chip containing 3.6 µL of medium per microwell. The sandwiched system was incubated in a humidity chamber to prevent evaporation of medium for the duration of a culture of up to 8 days (Supplementary Fig. 4). The relatively high density of cells of 1 × 10^7^ cells/mL on the pillar surface (Fig. 5b and Supplementary Fig. 5) enabled the formation of spheroids as the cells grew within the Matrigel matrix on the pillar surface. The spheroid morphology becomes particularly pronounced at 8 days of culture (Supplementary Fig. 5).

To ensure that the one among the many characteristics found in a TME was recapitulated, the expression of HIF1α was measured using confocal microscopy. A HIF1α antibody was used to stain MiaPaCa-2 spheroids encapsulated in Matrigel after they were cultured for 5 days. A nuclear stain (Hoechst 33342) was used to label all cells within the spot. MiaPaCa-2 spheroids stained positively for HIF1α indicating that the spheroids exhibited hypoxia (Fig. 5c, d). A cross-sectional view of the spheroids reveals that HIF1α is more highly expressed at the bottom and middle of the spheroid than at the top. Only fluorescent spots colocalized with the nuclear stain are cells. Any other stained spots are staining artifacts from cell debris. This is to be expected as both the bottom and middle of the spheroid have reduced access to oxygen in comparison to the cells at the top, which maintain direct contact with air. In addition, we did not observe any issues with dye penetration. This can best be seen in the cross-sectional view in Fig. 5c where there is uniform penetration of the nuclear dye Hoechst 33342. These results indicate that the high-density culture of target cancer cells can accurately represent the hypoxic conditions often found in a TME.

### High-content imaging of 3D tumor spheroid micropillar array for effector cell-mediated cytotoxicity including NK antibody/drug combination

Uniform-sized 3D spheroids in the 330-micropillar array were used to perform high-throughput ADCC on three different cancer cell lines; MiaPaCa-2, MCF-7, and MDA-MB-231, the latter a triple-negative breast cancer cell line, in the presence and absence of two chemotherapeutic drugs, doxorubicin and paclitaxel. The ADCC screen was performed by spotting 250 nL onto polydopamine-coated micropillars containing 2500 target cancer cells per spot. The cells were cultured for 3 days and then treated with NK92-CD16 cells. Three different E:T ratios (1:1, 5:1, and 10:1) were tested in the presence and absence of 2.5 µg/mL Trastuzumab, 3.5 nM doxorubicin, or 7.0 nM paclitaxel. The antibody concentration was chosen based on previous ADCC experiments in the 2D pillar/well sandwich platform and the drug concentrations were chosen to be ~tenfold lower than the EC_50_ values obtained from 2D cell culture for the three cell lines^47^. Combinatorial addition of both Trastuzumab and doxorubicin, as well as Trastuzumab and paclitaxel was also performed at all three E:T ratios. As a control, one block of spots was reserved for exposure of target cells with both antibody and the drugs but without NK92-CD16 cells. The micropillar/microwell sandwich platform was incubated for 24 h upside down to allow for target cell killing on culture day 3. The next day, the chips were then washed, stained with Hoechst 33342, Calcein-AM, and Propidium Iodide, and imaged using the ASFA^TM^ scanner.

**Fig. 6:**
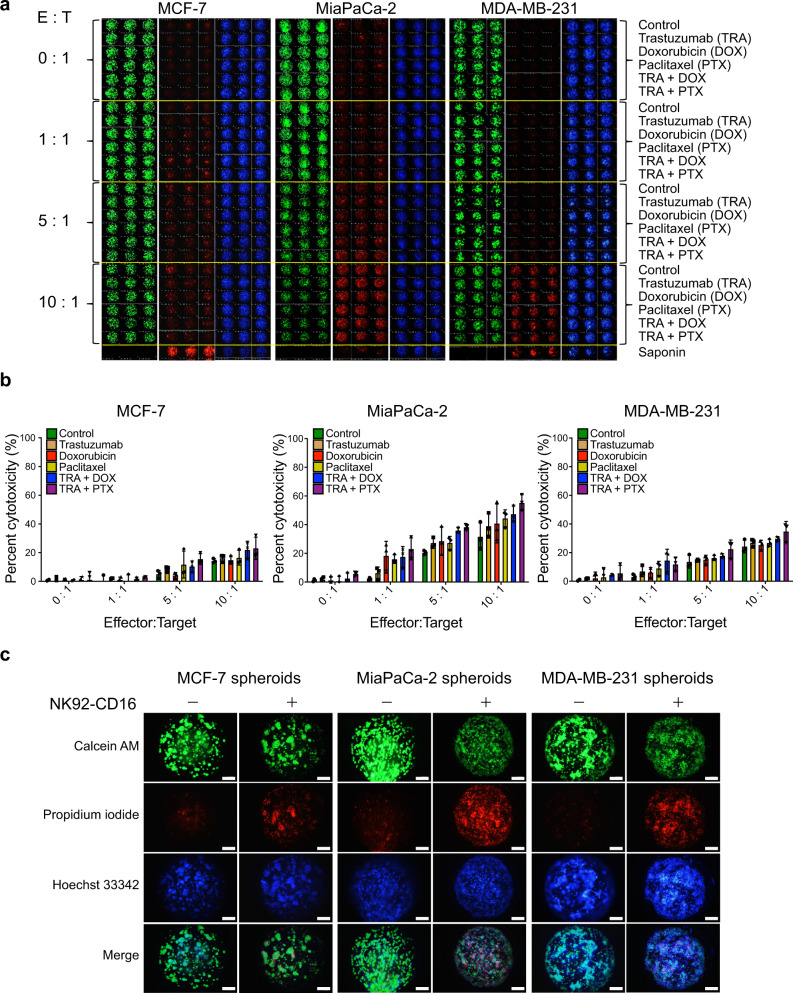
High-content images of 3D tumor spheroid micropillar array using the 330-micropillar/microwell chip sandwich platform. **a** Scanned images of the chip containing tumor spheroids including MCF-7, MiaPaCa-2, and MDA-MB-231 spheroids after treating NK92-CD16 cells combining with doxorubicin (DOX), paclitaxel (PTX), and Trastuzumab (TRA). **b** Cytotoxic activity of NK92-CD16 cells (effector) combining with DOX, PTX, and Trastuzumab was quantified by live 3D tumor spheroid (target) images stained with Calcein-AM. Percent cytotoxicity was calculated by NK cell-mediated killing/maximum killing by saponin. **c** Magnified images of 3D tumor spheroids after treating with NK92-CD16 cells (E:T = 10:1) in comparison with control (no treatment of NK92-CD16 cells). Error bars indicate mean ± SD for *n* = 3. Scale bars show 200 µm.

For all three cell lines, increasing the E:T ratio from 1:1 to 10:1 increased target cell cytotoxicity (Fig. 6a, b). As the number of NK92-CD16 cells added was increased, they were more effective in killing target cancer cell spheroids. The combination of NK92-CD16 at the E:T ratio of 10:1, Trastuzumab, and doxorubicin or paclitaxel was significantly more effective in target cell cytotoxicity (*p* < 0.01 or <0.05 for doxorubicin or paclitaxel, respectively) for all three cell lines. In addition, there were minimal differences in cytotoxic responses of the three cancer cell lines among the antibody only, the drug only, and combined antibody-drug conditions at all three E:T ratios when the cancer cells were incubated for 24 h. This indicates that the primary contributor for cell death in target cells in the presence of NK92-CD16 cells. In addition, among the three cell lines tested, MiaPaCa-2 spheroids exhibited the greatest cytotoxicity in response to NK92-CD16 exposure, followed by MDA-MB-231 and then MCF-7 spheroids (Fig. 6b, c and Supplementary Data 4). This is contrary to what was observed in 2D monolayer culture where MCF-7 cells were found to be more likely to die upon NK92-CD16 exposure than MiaPaCa-2 cells. Statistical analysis was performed for each cell line tested using ANOVA followed by a post hoc Dunnett’s test (Supplementary Fig. 6). Finally, the presence of residual NK92-CD16 cells did not affect target cell viability. Specifically, NK92-CD16 cells were pre-stained with CellTracker Red prior to their addition to the 330-microwell chip and staining by calcein and CellTracker Red was visualized (Supplementary Fig. 7 and Supplementary Data 5). The majority of NK92-CD16 cells were washed away prior to staining, very little CellTracker Deep Red staining was observed with all three target cancer cell lines.

**Fig. 7:**
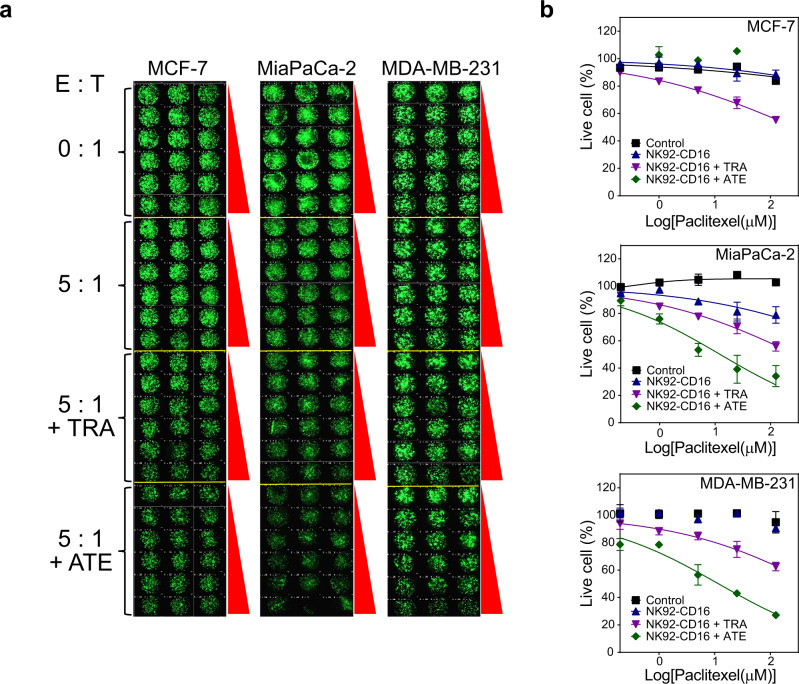
High-content imaging analyses for NK-CD16 cell-mediated killing against MCF-7, MiaPaCa-2, and MDA-MB-231 tumor spheroids. **a** Scanned images of live 3D MCF-7, MiaPaCa-2, and MDA-MB-231 tumor spheroid microarrays after treating with various concentrations of paclitaxel (PTX), NK92-CD16 cells (effector, E), Trastuzumab (TRA), and Atezolizumab (ATE). **b** Dose-responsive curves for PTX with/without TRA and ATE in the presence/absence of NK-CD16 cells. Error bars indicate mean ± SD for *n* = 3.

Since all three cell lines displayed increased cell death upon combinatorial exposure of NK92-CD16 with doxorubicin or paclitaxel and Trastuzumab, we expanded to a second antibody, Atezolizumab, an anti-programmed death-ligand 1 (PDL1) antibody that acts as a checkpoint inhibitor^48^. Dose-response behavior of the three target cell lines was observed upon exposure to Atezolizumab plus paclitaxel at the same NK92-CD16 ratio (Fig. 7a, b and Supplementary Data 6). We were interested in whether MDA-MB-231, which is a triple-negative breast cancer cell line having reduced expression of estrogen receptor (ER), progesterone receptor, and HER2^49^, was susceptible to cell death upon combination exposure of paclitaxel, Atezolizumab, and NK92-CD16 cells. Triple-negative breast cancers are particularly aggressive to treat due to very low expression levels of ER, PR, and HER2^50^. To test this, the three target cancer cell lines were spotted onto the 330-micropillar platform to generate the corresponding 3D spheroids. A range of paclitaxel concentrations were used in the presence and absence of NK92-CD16, Trastuzumab, and Atezolizumab. The 3D spheroids on the micropillar chip were incubated for 24 h, and then stained and imaged. The killing of MCF-7 tumor spheroids was augmented by the presence of the Trastuzumab and NK92-CD16 cells, presumably due to HER2 antibody-dependent NK cell-mediated cytotoxicity (Fig. 7a, b). In the absence of the effector with NK92-CD16 cells, minimal paclitaxel-induced cytotoxicity was observed (Supplementary Table 1) with EC_50_ values >500 µM. For MCF-7, the most cytotoxic condition was obtained with paclitaxel in the presence of both Trastuzumab and NK92-CD16 cells; ~1.8-fold lower EC_50_ value than in the absence of the antibody and effector cells. Interestingly, the same condition against MDA-MB-231 spheroids did not prove to be effective. Rather, an ~43-fold lower EC_50_ value was obtained for paclitaxel in the presence of Atezolizumab along with NK92-CD16 cells. This is not surprising since MDA-MB-231 cells express high levels of PDL-1^51^, and hence, an antibody targeting this receptor should be able to augment the killing of MDA-MB-231 cells in the presence of paclitaxel. For MiaPaCa-2 spheroids, both antibodies were effective in inducing cytotoxicity with Atezolizumab performing substantially better than Trastuzumab. Finally, paclitaxel in combination with either antibody alone (in the absence of NK92-CD16) did not elicit the same cytotoxic response in target spheroids (Supplementary Fig. 8, Supplementary Table 1, and Supplementary Data 7). This implies that the presence of NK92-CD16 cells were crucial to ensuring target spheroid cell killing.

## Discussion

A 330 micropillar-microwell platform was developed to enable cancer-immune cell coculture to be used in the rapid, high-throughput screening of ADCC for cancer immunotherapy. The micropillar platform was effective in multiple designs for use in ADCC screening of NK-cell activity against a human pancreatic ductal adenocarcinoma cell line (MiaPaCa-2) and a human breast adenocarcinoma cell line (MCF-7). Specifically, a simple 2D cancer cell monolayer could be extended to a 3D model with the cells embedded in Matrigel, and then to uniform-sized tumor spheroids. The latter provides conditions for screening in a contextually relevant 3D microenvironment. Differences in NK92-CD16 mediated cytotoxicity were evident between 2D and 3D environments. For example, while MCF-7 cells exhibited high levels of cell death due to ADCC in 2D, the same cells were much more resistant to NK92-CD16 cells when they were cultured as 3D spheroids. Furthermore, both MiaPaCa-2 and MCF-7 exhibited ~20% cytotoxicity in 2D upon the addition of Trastuzumab in the absence of NK92-CD16 cells. Yet this behavior was virtually eliminated when the same cells were cultured in 3D format. This may be due to the acquired resistance of MCF-7 cells when they were cultured in 3D spheroids. Previous work has shown that the expression of HIF1α in MCF-7 contributes to its resistance to chemotherapeutic drugs including doxorubicin^52^. In addition, when certain breast cancer cells were cultured under hypoxic conditions in 3D, they were found to acquire resistance to Trastuzumab through changes in the HER2 expression causing a diminished response to the antibody^43^. MiaPaCa-2 cells also display higher basal levels of MMPs than MCF-7^53,54^. The presence of MMPs in cells can cause them to become highly metastatic and invasive. Indeed, MiaPaCa-2 cells have the ability to penetrate Matrigel using Transwell Invasion assay^55^. This implies that a similar process may occur in our micropillar-microwell system, wherein the degradation of the Matrigel surrounding the target cells may provide easier access for NK92-CD16 cells to infiltrate the 3D structure. This increased access produces higher cytotoxicity against MiaPaCa-2 cells than against MCF-7 cells that do not express high basal levels of MMPs. Similarly, MDA-MB-231, a highly invasive breast cancer cell line^56^, has a high expression of MMP9^57^. This could explain the observed higher cytotoxicity behavior in MDA-MB-231 spheroids in comparison to MCF-7 spheroids.

The presence of an ECM can limit the cytotoxicity of drugs and antibodies, and can play a major role in determining the response of cancer to a particular treatment^58^, and such response may be poorly predictive using conventional 2D in vitro models. The micropillar-microwell platform, particularly under conditions that mimic the TME, therefore, has the potential to serve as a useful tool for early-stage immunotherapy discovery.

The poor response of conventional anticancer drugs, such as paclitaxel and doxorubicin, used without ADCC, is not surprising since multicellular spheroids (MCS) can be highly resistant to drug exposure^59^. In our case, the observed poor response is not a result of inflated cell viability measurements due to cancer cells leaving the 3D matrix, as the pillars were incubated face up during the NK92-CD16 exposure and we did not observe the target cancer cells in the well chip. This may be because the cancer cells are mixed with liquid Matrigel prior to undergoing gelation within the spot and becoming encapsulated within the matrix. Furthermore, the mechanisms used by cancer cells and immune cells for migration may be different. NK cells, like other immune cells, utilize ameboid migration by reorganizing their cytoskeleton. This is typically faster than mesenchymal migration, which requires matrix degradation using proteases. This would explain why NK cells migrate but the target cells do not. In addition, cells within the MCS have varying responses to the drug depending upon where they are located within the spheroid. For example, cells at the center of the spheroid were found to be more than 90% viable even after 3 days of drug exposure^60^, presumably because of very low or no cell growth deep within the spheroid structure. It should be noted that the cytotoxicity of paclitaxel on different cancer cell lines has been determined primarily in terms of growth inhibition over 3 days or more^60–62^. In our case, the EC_50_ value is a measure of paclitaxel concentration at which NK92-CD16 cells cause apoptosis of the MCS in the presence of drugs and antibodies over a shorter 24-h time period. Prolonged exposure of the cancer cells to paclitaxel in the MCS would likely be required to cause apoptosis. Finally, hypoxic MCS has been shown to be highly resistant to drug exposure^52,62^. Since our platform can be used to generate spheroids with hypoxic conditions in the interior of the MCS, high EC_50_ values are to be expected.

The resistance of cancer cells to several chemotherapeutic drugs may be overcome by coupling them to immunotherapeutics. Although paclitaxel alone was poorly effective as a cytotoxic agent against both MCF-7 and MDA-MB-231 cells in spheroid cultures, the addition of NK92-CD16 cells with Trastuzumab increased cytotoxicity. Indeed, combinatorial treatments have been shown to be more successful in clinical trials for breast cancer patients than drug or antibody treatment alone^63^. The synergistic effects of antibodies, small molecule drugs, and NK cells have been reported previously for multiple different cancer cell lines^64^. Specifically, a synergistic behavior with Trastuzumab and paclitaxel was observed in MCF-7 spheroids along with NK92-CD16 cells (IC_50_ of paclitaxel alone 28.2 nmol/L)^65^. Such synergism has been attributed to immune cell recognition and targeting of cancer cells upon release of stress-associated factors (i.e., heat shock proteins (HSPs), high-mobility group box 1 proteins (HMGB1s), etc.) in the presence of chemotherapeutic drugs^66^. In addition, reorganization of cell surface receptors upon addition of cytotoxic drugs is known to make cancer cells more vulnerable to immune cell-mediated cell death^67^. Therefore, our platform appears to recapitulate the complex interactions that occur between immune cells and therapeutics within the TME. The presence of NK92-CD16 cells was able to accelerate the process of cytotoxicity to 24 h in combination with the drugs. This is particularly relevant as previous reports have shown that breast cancer cells can exhibit short-term resistance during drug exposure^61^. Therefore, our platform may be used to screen and identify immunotherapy combinations to treat highly aggressive forms of cancers. One caveat to this concept is the potential for NK92-CD16 cell metabolism to indirectly cause some target cell death, i.e., killing not due to NK-specific action. However, the ratio of NK92-CD16 cells to target cancer cells was within the range of that reported in the literature for cancer 3D spheroid cultures^26,68^.

Finally, Atezolizumab is an antibody that lacks the Fc receptor and is not ideal for ADCC. Nevertheless, we show that the presence of the antibody can potentiate cytotoxicity. This may be due to the PDL-1 blockade on the surface of cancer cells. Since, Atezolizumab is an anti-PDL1 antibody, it can bind to the PDL-1 on the cancer cell surface. This prevents PDL-1 interaction with the corresponding PD-1 receptor found on the NK cell surface^69,70^. The lack of this interaction prevents the cancer cells from undergoing immune escape and enhances NK92-CD16 killing of target cells^70^.

To effectively evaluate these myriad combination approaches, a platform is needed that allows for simple and facile measurement of cell viability post-addition with immune cells, antibody, and small molecule chemotherapeutic drugs. The current standard for measuring immune (effector) mediated cytotoxicity uses either chromium release or calcein assays^24,26^. Both methods require pretreatment of target cells with a specific dye. The platform described herein enables easy removal of effector cells after exposure with target cells, and hence, prestaining is unnecessary. While we tested 25 conditions per chip, more than 50 different conditions can be identified using one single micropillar-microwell platform system with three or more replicates allowing for rapid testing of a large candidate library.

In conclusion, a 330-micropillar/microwell sandwich platform was developed that enables the coculture of both immune cells and various 3D cancer spheroids. The platform faithfully recapitulated the hypoxic environment that is often present within 3D tumor spheroids and that makes target cells more resistant to certain therapeutics. In addition, since NK92-CD16 cells used in these experiments were modified to express the CD16 receptor, the cells have the ability to use the fragment crystallizable region (FcR)-mediated immune (effector) engagement to induce ADCC against multiple different cancer cell lines. A wide variety of therapeutic drugs and antibodies have been developed with mixed success, and this is partly due to the highly personalized nature of cancers^71^. The platform described herein has the potential to be adapted to culturing patient-derived cancer cells and primary immune cells. Specifically. the 330-micropillar/microwell sandwich platform can help identify combination therapies that work best against a patient’s specific cancer. Hence this tool may serve to aid the identification and delivery of personalized immunotherapy.

## Methods

### Cancer cell and NK92-CD16 culture

MiaPaCa-2 cells (ATCC CRL-1420) were cultured in Dulbecco’s Modified Eagle Medium (DMEM) supplemented with 10% fetal bovine serum (FBS) and 1% Penicillin-Streptomycin (Pen-Strep). Materials are provided in the Supplementary Information. MCF-7 cells (ATCC HTB-22) were cultured in DMEM/F12 supplemented with 10% FBS, 0.01 mg/mL insulin, 2 mM Glutamax, and 1% Pen-Strep. MDA-MB-231 (ATCC HTB-26) cells were cultured in DMEM (high glucose) supplemented with 10% FBS and 1% Pen-Strep. NK92-CD16 cells (ATCC PTA-6967) were cultured in Prime XV NK Cell Chemically Defined Medium (Irvine Scientific, Irvine, CA) supplemented with 100 U/mL of IL-2 (PeproTech, Cranbury, NJ) and 1% Pen-Strep. The cancer cells were grown in T75 flasks and NK cells were grown in 24-well and 6-well ultra-low attachment plates. The cells were all maintained in a 37 °C incubator at 5% CO_2_. The cancer cells were passaged when they reached 70–80% confluent. The NK cells were passaged when they reached 1 × 10^6^ cells/mL and the medium was exchanged every 2 days.

### 2D and 3D experiments on 384-pillar/well sandwich system

Polystyrene 384 pillar plates (MBD Korea Co., South Korea) were used for both 2D and 3D cell culture experiments. For 2D experiments, the pillar plates were coated with 1% fibronectin from bovine plasma by stamping the pillar plates on 384 well plates containing 40 µL of 1% fibronectin in DPBS. The sandwiched plates were placed in 37 °C for 1 h. The pillar plates were then removed from the solution and stored at 4 °C until they were ready for printing. For cell printing, target cancer cells were washed with Dulbecco’s Phosphate Buffered Saline (DPBS), detached with 0.25% Trypsin-EDTA and spun down at 150 × *g* for 5 min. The cells were resuspended in a fresh medium at a concentration of 2.5 × 10^6^ cells/mL. Cell suspension (1 µL) was spotted on fibronectin-coated plates using an ASFA^TM^ fifth generation cell spotter (MBD Korea Co., South Korea). The plate was then incubated in a humidity chamber with pillars facing up at 37 °C for 6 h to allow for cell attachment. The pillar plate was then sandwiched with a conventional 384-well plate containing fresh medium. After 2–3 days, NK92-CD16 cells were added with or without 1 and 5 µg/mL Trastuzumab in 384-well plates. The pillar plates containing target cells were stamped with the well plates containing NK cells and incubated upside down overnight. The next day the pillar plates were stained in 4 µM Calcein-AM and 5 µg/mL Hoechst 33342 diluted in fresh medium for 30 min at 37 °C. The plate was then imaged using a Cellomics ArrayScan XTI (Thermo Fisher Scientific, Waltham, MA). Image analysis for quantifying fluorescent intensities and merging images were performed using ImageJ software (NIH)^72^. The region encompassing the pillar spot was selected using ImageJ. The measure function on ImageJ was used to obtain mean fluorescence intensity in relative fluorescence units (RFU) for each spot in the green channel. The percent cytotoxicity was then normalized based on calcein-AM area and calculated using Eq. 1,

1 $$\% {{{{\mathrm{Cytotoxicity}}}}}=1-\frac{{{{{{\mathrm{Fluorescence}}}}}\;{{{{\mathrm{intensity}}}}}}_{{{{{\mathrm{Untreated}}}}}\,{{{{\mathrm{cells}}}}}}-{{{{\mathrm{Fluorescence}}}}}\,{{{{{\mathrm{intensity}}}}}}_{{{{{\mathrm{Treated}}}}}\,{{{{\mathrm{cells}}}}}}}{{{{{{\mathrm{Fluorescence}}}}}\;{{{{\mathrm{intensity}}}}}}_{{{{\mathrm{Untreated}}}}\,{{{{\mathrm{cells}}}}}}-{{{{\mathrm{Fluorescence}}}}}\,{{{{{\mathrm{intensity}}}}}}_{{{{{\mathrm{Saponin}}}}}\,{{{{\mathrm{treated}}}}}\,{{{{\mathrm{cells}}}}}}}$$

where untreated cells were not exposed to NK92-CD16 cells or drugs or antibodies, and the saponin-treated cells served as the dead control.

For 3D cell culture, 384 pillar plates were first coated with polydopamine by incubating in a solution containing 2 mg/mL dopamine hydrochloride in Tris HCl at pH 8.5 for 2 h on a room temperature shaking incubator at 120 rpm. The plates were washed with DI water, dried, and stored until further use. The dopamine coating on the surface of the pillar enhances the attachment of Matrigel to the pillar surface due to the covalent binding between the polydopamine coating and the proteins in the Matrigel^73^. We have previously coated chips with dopamine hydrochloride to culture cells in 3D without any issue^74^. The cancer cells were harvested and mixed with high concentration growth factor reduced Matrigel (Basement membrane extracted from Engelbreth–Holm–Swarm mouse sarcoma, Corning Life Sciences Catalog # 354263) to a final cell concentration of 2.5 × 10^6^ cells/mL for pillar plate. Matrigel-cell mixture (1 µL) was then spotted using the precooled ASFA^TM^ spotter onto coated pillar plates, which were then incubated face down at 4 °C for 15 min to allow for cell aggregation at the bottom of the spots. The plates were then placed at 37 °C in a humidity chamber to allow for spot gelation. The pillar plates were subsequently sandwiched with well plates containing fresh medium.

### 3D experiments on 330-micropillar/microwell sandwich system

Polystyrene 330-micropillar chips (MBD Korea Co., South Korea) were coated with polydopamine, washed, and dried until further use. The 330-well chips were UV-treated using a 96 W transilluminator (Syngene GVM-30) for 4 h to enhance surface hydrophilicity. Cancer cells were detached and mixed with high concentration growth factor-reduced Matrigel to obtain a final cell concentration of 1 × 10^7^ cells/mL. Then, 250 nL of the Matrigel-cell mixture was spotted using the ASFA^TM^ spotter onto the polydopamine coated 330-micropillar chips. The chips were incubated face down in a humidity chamber at 37 °C for 15 min to allow for gelation and then they were sandwiched with the corresponding 330-microwell chip containing 3.6 µL of fresh medium per well. The chips were cultured for a period of 3 days prior to NK cell exposure. The cell growth medium on the chip was exchanged every 2 days. For cytotoxicity experiments, where the NK92-CD16 ratio was varied, 25 different conditions were tested (Supplementary Table 2), including a dead control (via the addition of saponin). Similarly, for the dose-response experiments with paclitaxel, where the E:T ratio was fixed at 5:1, 25 different conditions including a dead cell control were evaluated (Supplementary Table 3). An unconjugated anti-HER2 antibody was used for experiments on 330-micropillar/microwell chips. The NK92-CD16 cells, Trastuzumab, and the drugs were spotted on a 330-microwell chip. The micropillar chip with target cells was then stamped onto the 330-microwell chip and incubated upside down for 24 h. The micropillar chips were then washed with 8 mL DPBS added to a 4-well rectangular dish and stained with 4 µM Calcein AM (for live-cell staining), 10 µg/mL propidium iodide (for dead-cell staining), and 5 µg/mL Hoechst 33342 (for nuclear staining) in 8 mL of DPBS with 1 g/L d-glucose for 30 min. The chips were subsequently washed in DPBS and imaged using an ASFA^TM^ Cell Scanner (MBD Korea Co., South Korea) and quantitatively analyzed using cell analyzer software within the imaging system. The cell analyzer software provided quantitative values for the mean area of the fluorescence for each individual spot. The % cytotoxicity was then calculated using Eq. 1 except with fluorescence mean area instead of fluorescence intensity.

### Immunofluorescence

Immunofluorescence was performed on MiaPaCa-2 3D spheroids to measure the expression of hypoxia-inducing factor 1α (HIF1α). To generate spheroids, 250 nL of Matrigel-cell mixture (at a concentration of 1 × 10^7^ cells/mL) was spotted on a glass bottom confocal dish using the ASFA^TM^ spotter. The spots were gelled by incubating the dish upside down in a humidity chamber for 15 min at 37 °C. Fresh DMEM was added to the confocal dish to cover the spots and the spheroids were cultured for 5 days, and then the spheroids were washed with DPBS and fixed with 4% (w/v) paraformaldehyde and 0.25% glutaraldehyde (w/v) in DPBS for 20 min. The spheroids were permeabilized with 0.25% (v/v) Triton X-100 in DPBS for 30 min and quenched with 2 mg/mL sodium borohydride in DPBS. The spheroids were then blocked with 5% (w/v) bovine serum albumin and 1% (v/v) goat serum in DPBS overnight at 4 °C and stained with mouse 10 µg/mL anti-HIF1α antibody (R&D systems MAB1536) in 1% (w/v) BSA in DPBS overnight at 4 °C. An Alexa Fluor 488 goat anti-mouse (Invitrogen A28175) antibody was used as a secondary antibody with staining done in 1% BSA in DPBS overnight at 4 °C at 1:500 dilution. The cells were then stained with 5 µg/mL Hoechst 33342 for 10 min at room temperature and imaged using a Leica TCS SP8 STED Confocal Microscope. Z-stacks of the spheroids were constructed using Imaris Viewer.

### Cell viability and apoptosis

Confocal microscopy was performed on MCF-7 aggregates in 3D to evaluate NK92-CD16 cytotoxicity with Calcein AM staining and triple color staining. For Calcein-AM staining, MCF-7 cells were mixed with high concentration growth factor-reduced Matrigel at a final concentration of 2.5 × 10^6^ cells/mL and spotted onto a glass bottom confocal dish. The 3D aggregates were cultured for 5 days and then stained with 4 µM Calcein-AM and 5 µg/mL Hoechst 33342 diluted in DPBS with 1 g/L d-glucose for 30 min at 37 °C. For triple color staining with Cell Event Caspase 3/7 Green, Cell Tracker Deep Red, and Hoechst, MCF-7 cells were spotted on glass bottom confocal dish as before. NK92-CD16 cells were pre-stained with 1 µM Cell Tracker Deep Red prior to their addition to MCF-7 aggregates. The stained NK92-CD16 cells were added to the MCF-7 aggregates and incubated for 1 and 24 h. The MCF-7 aggregates were then stained with 4 µM Cell Event Caspase 3/7 Green and 5 µg/mL Hoechst 33342. The aggregates were subsequently imaged using Leica TCS SP8 STED Confocal Microscope at 40X magnification with 2 µm z-stack width. Image analysis was carried out using an Imaris 9.7 cell analyzer. Volume reconstruction of a cross-sectional view of the spheroids was constructed for each channel.

### Flow cytometry

For detection of HER-2 expression using flow cytometry, 1 × 10^6^ MCF-7 and MiaPaCa-2 cells were detached using 0.25% Trypsin EDTA and washed with DPBS. The cells were then stained with 10 µg/mL of Alexa Fluor 488 conjugated Anti-HER2 antibody diluted in 1% (w/v) BSA in DPBS. The cells were then washed three times in DPBS to remove any unbound antibody and then submitted to flow cytometry (BD LSR II). A negative control that was not stained was also run to establish the fluorescence gates. The results were then analyzed using Flow Jo v7.6.

### Statistics and reproducibility

All results were analyzed using GraphPad Prism. The error bars on figures indicate mean ± standard deviation (SD) unless otherwise indicated. Three replicates for each condition were performed for each cell type. For multiple pairwise comparisons (between different effector:target ratio), a one-way ANOVA was used to determine statistical significance for *n* = 3. A post hoc Dunnett’s test was performed following ANOVA to identify which groups were statistically different from each other. For the 2D experiments comparing the effect of Trastuzumab vs. no Trastuzumab, an unpaired student’s *t*-test was used. A *p* < 0.05 was considered significant in both cases. For dose-response curves without clearly defined top or bottom plateaus, these values were set to either the maximum or minimum viability values within the sampled range^75^. The sigmoidal dose-response curves and EC_50_ values for a drug compound were obtained using Eq. 2:

2 $${{{\rm{Y}}}}={{{\rm{Bottom}}}}+\frac{\left({{{\rm{Top}}}}-{{{\rm{Bottom}}}}\right)}{1+{10}^{\left({{{\rm{LogEC}}}}50-{{{\rm{X}}}}\right)\ast {{{\rm{H}}}}}}$$

where EC_50_ is the midpoint of the curve, H is the hill slope, X is the logarithm of a drug concentration, and Y is the response (% live cells), starting at the bottom and going to top with a sigmoid shape. The log(EC_50_) values were obtained from the dose-response curves generated in GraphPad Prism.

### Reporting Summary

Further information on research design is available in the Nature Research Reporting Summary linked to this article.

## Supplementary information

Supplementary Information

Supplementary Data 1

Supplementary Data 2

Supplementary Data 3

Supplementary Data 4

Supplementary Data 5

Supplementary Data 6

Supplementary Data 7

Reporting Summary

Description of Supplementary Files

## Data Availability

The datasets generated during and that support the findings of this study are provided as Supplementary Data 1–7.
