# Supplementary material for: 3D tumor spheroid microarray for high-throughput, high-content natural killer cell-mediated cytotoxicity
Source: Commun Biol. 2021 Jul 21;4:893. doi: 10.1038/s42003-021-02417-2 (PMC8295284; doi:10.1038/s42003-021-02417-2)
Supplement: Supplementary file 1 — Supplementary Information [file 42003_2021_2417_MOESM1_ESM.pdf]

Supplementary Information for

3D Tumor Spheroid Microarray for High-Throughput, High-Content NK Cell-Mediated Cytotoxicity

Sneha Gopal[1], Seok-Joon Kwon[1*], Bosung Ku[2], Dong Woo Lee[3], Jungeun Kim[2], and Jonathan Dordick[1*]

[1]Department of Chemical and Biological Engineering, and Center for Biotechnology & Interdisciplinary Studies, Rensselaer Polytechnic Institute, 110 8th Street, Troy, NY 12180, USA.

[2]MBD (Medical & Bio Decision) Co., Ltd. Suwon-si, Gyeonggi-do, 16229, Republic of Korea.

[3]Department of Biomedical Engineering, Konyang University, Daejeon, Republic of Korea.

[4]Departments of Biomedical Engineering and Biological Sciences, Rensselaer Polytechnic Institute, 110 8th Street, Troy, NY 12180, USA.

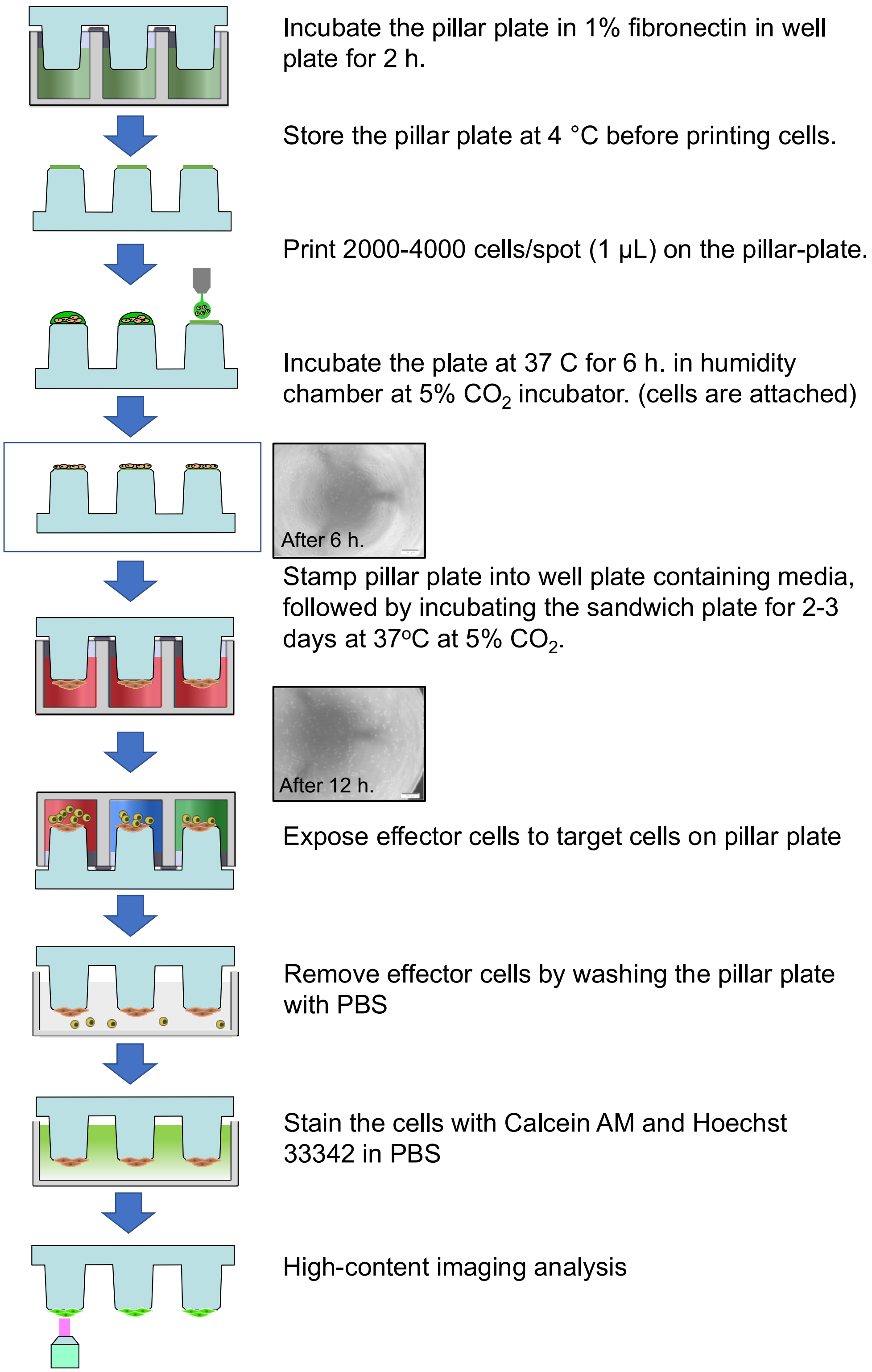

Supplementary Figure 1. Experimental protocols of 2D cancer cell culture on 384-pillar plate, followed by high-content imaging for NK-mediated cell cytotoxicity assay. Scale bars in the images are 200 μm.

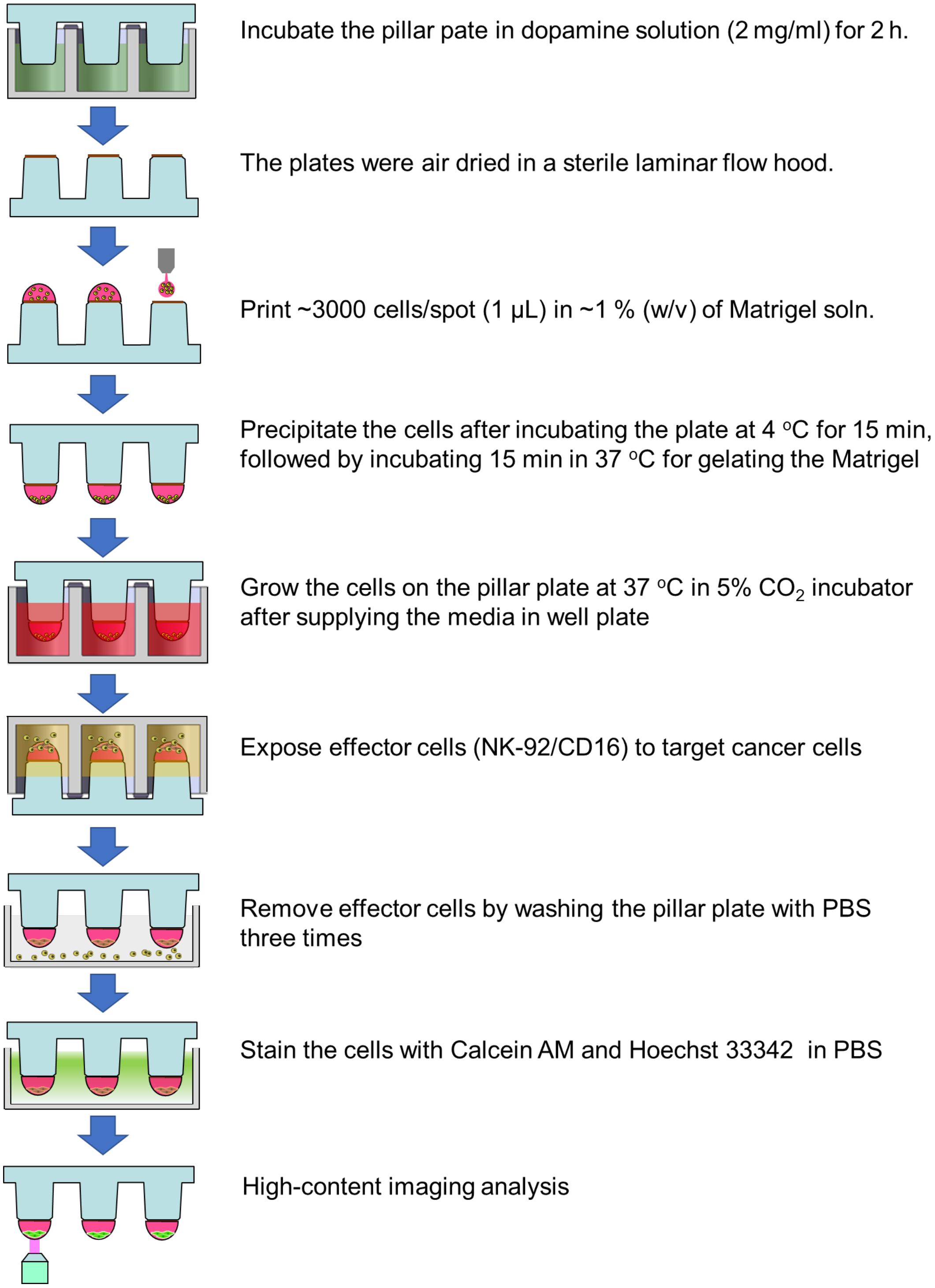

Supplementary Figure 2. Experimental protocols of 3D cancer cell culture on 384-pillar plate using Matrigel hanging drop method, followed by high-content imaging for NK-mediated cell cytotoxicity assay.

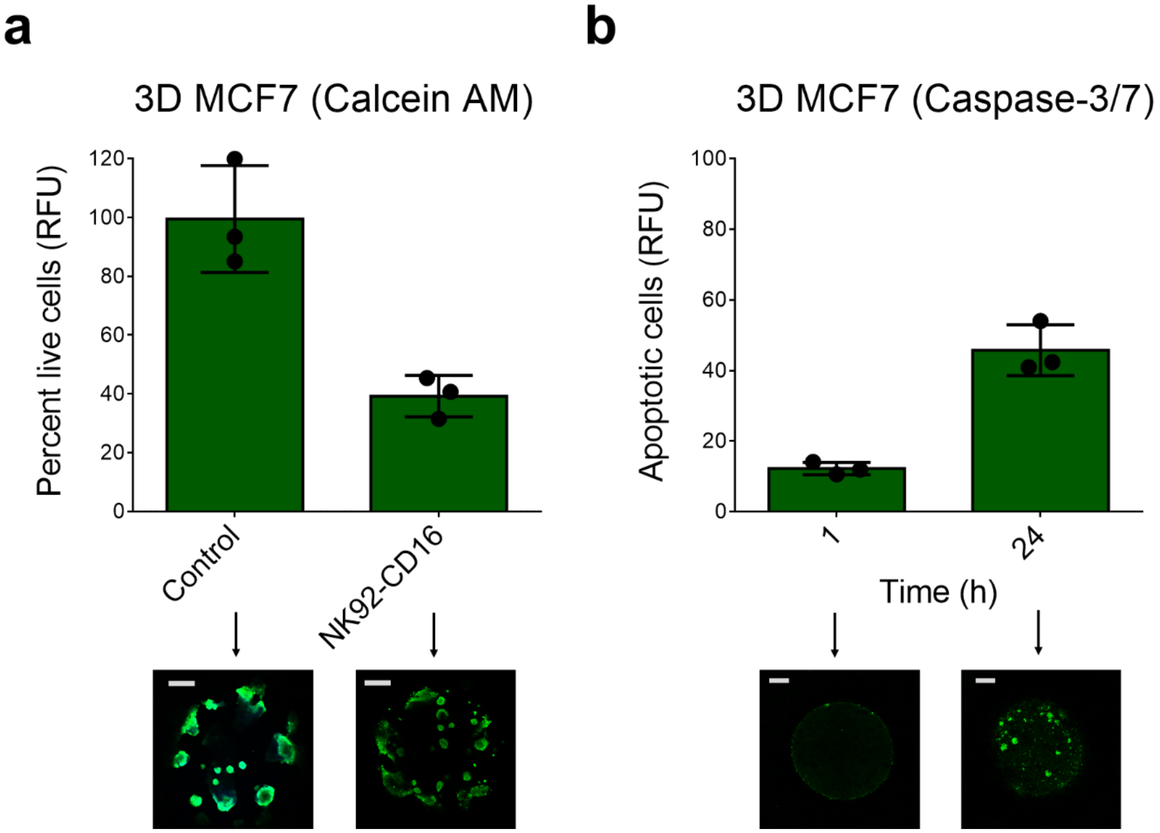

Supplementary Figure 3. Cytotoxicity of NK92-CD16 cells against 3D MCF-7 cells staining with Calcein-AM (a) and Caspase-3/7 (b). Three different representative regions were chosen in the images and selected using the rectangular select tool on ImageJ. The mean fluorescence intensity in relative fluorescence units (RFU) in the region was quantified for all images within those regions. The resulting values were then plotted using GraphPad Prism for both calcein and caspase 3/7 staining. The error bars represent standard deviation for ImageJ measurements across three different regions on the image. Scale bars show 200 μm.

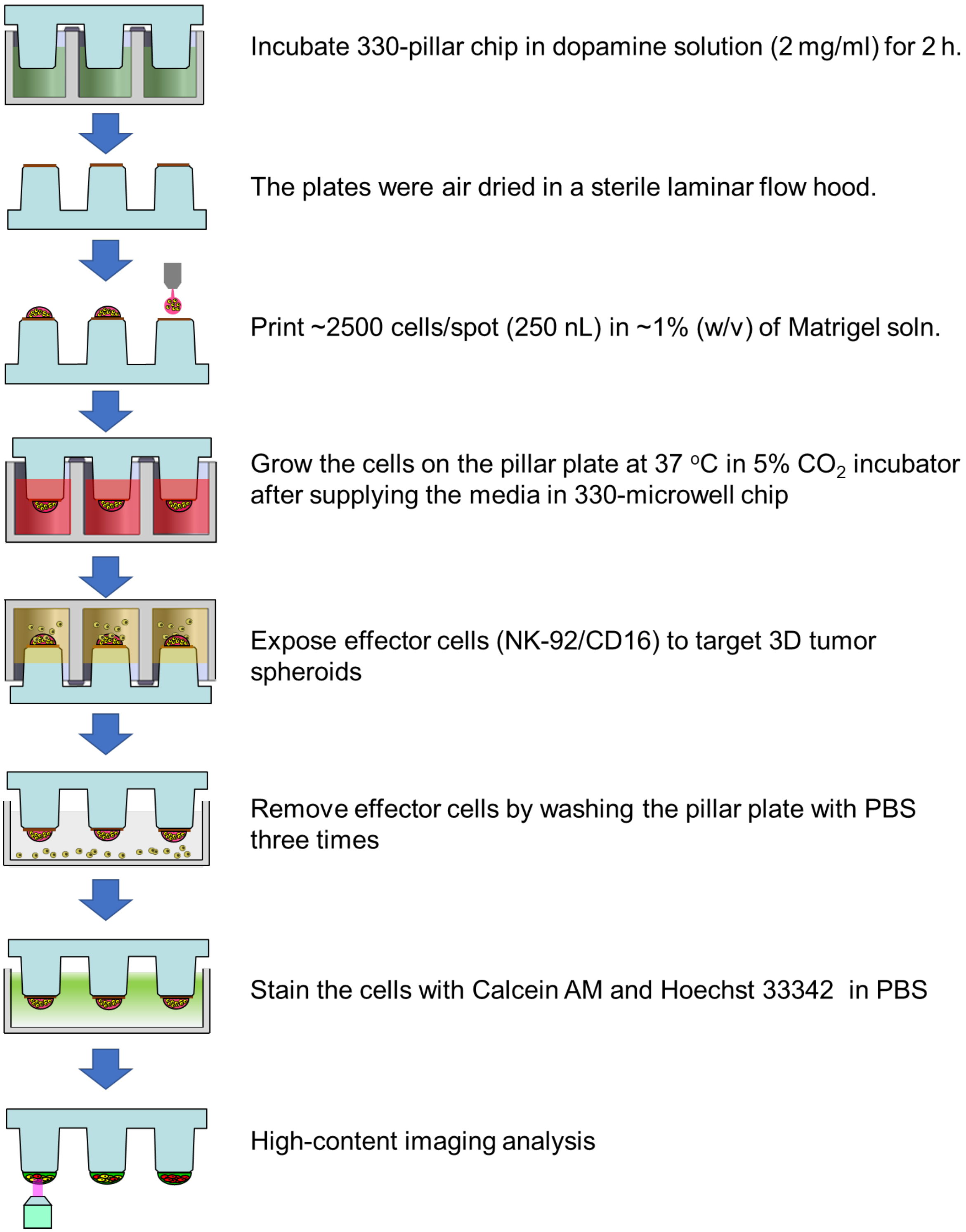

Supplementary Figure 4. Experimental protocols for 3D tumor spheroid micropillar array on 330-micropillar chip after printing high-density cells (2500 cells) in tiny volume (250 nL) of Matrigel solution, followed by high-content imaging for NK-mediated cell cytotoxicity assay.

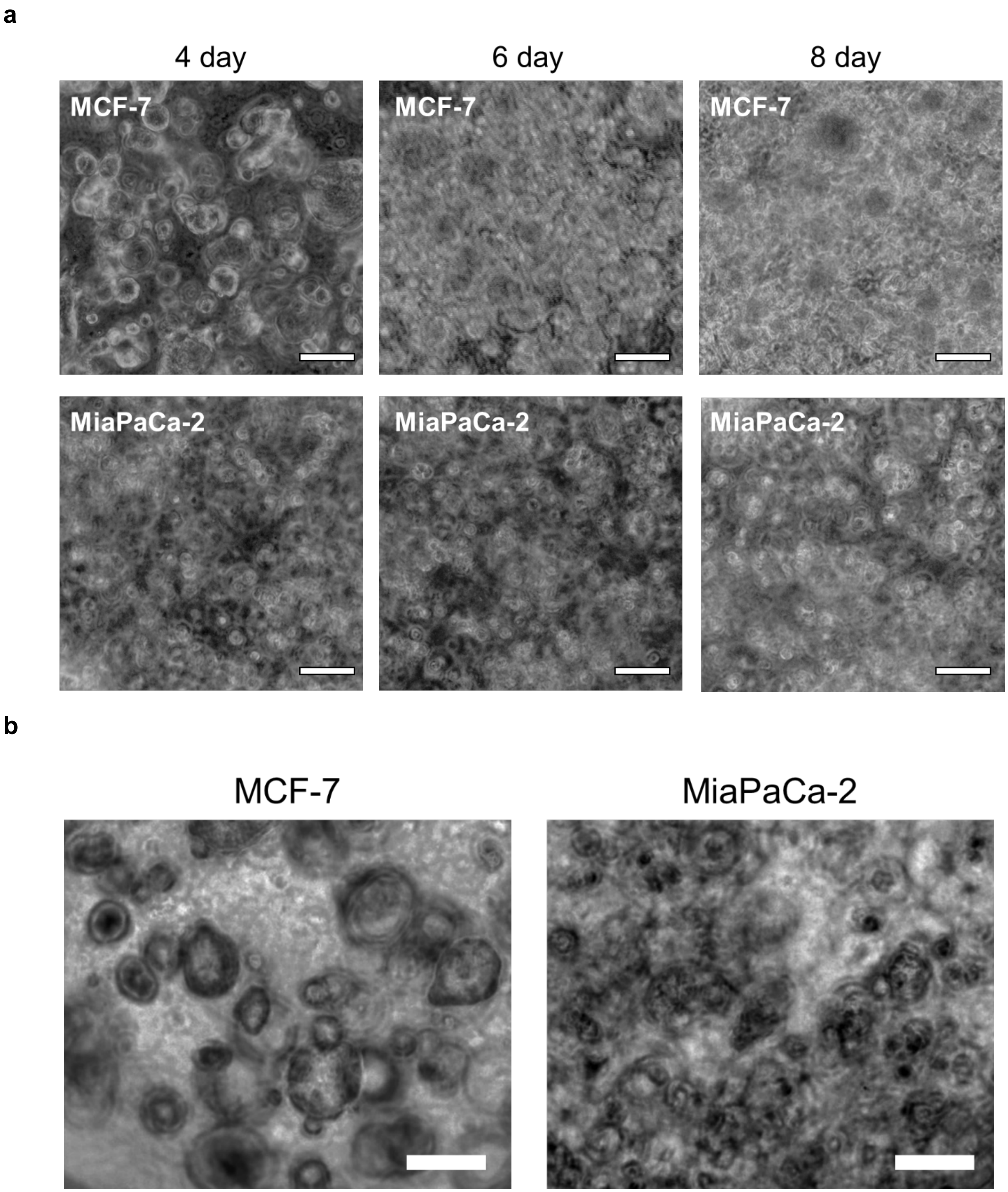

Supplementary Figure 5. (a) Bright field images of 3D MCF-7 and 3D MiaPaCa-2 for generating spheroids. Scale bars show 100 μm. Spheroid morphology becomes particularly pronounced as time increases. (b) Morphology of 3D MCF-7 (4 days) and 3D MiaPaCa-2 (6 days) spheroids in Matrigel using phase-contrast microscopy. Scale bars show 50 μm.

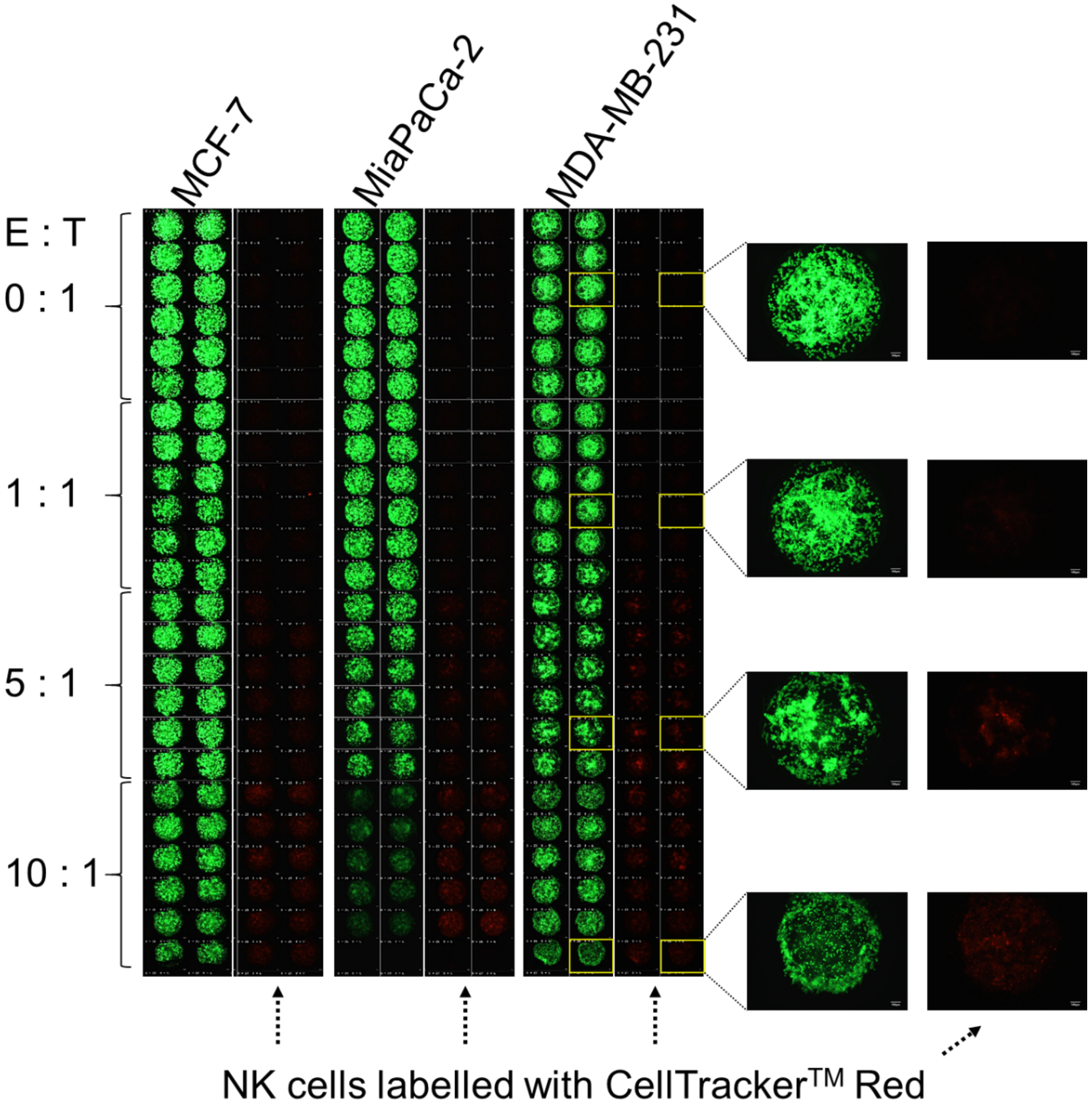

Supplementary Figure 6. Scanned images of the chip containing tumor spheroids including MCF-7, MiaPaCa-2, and MDA-MB-231 spheroids after treating NK92-CD16 cells labelled with CellTracker^{TM} Red. E and T represent effector (NK92-CD16 cells) and target tumor spheroids, respectively. Scale bars show 100 μm.

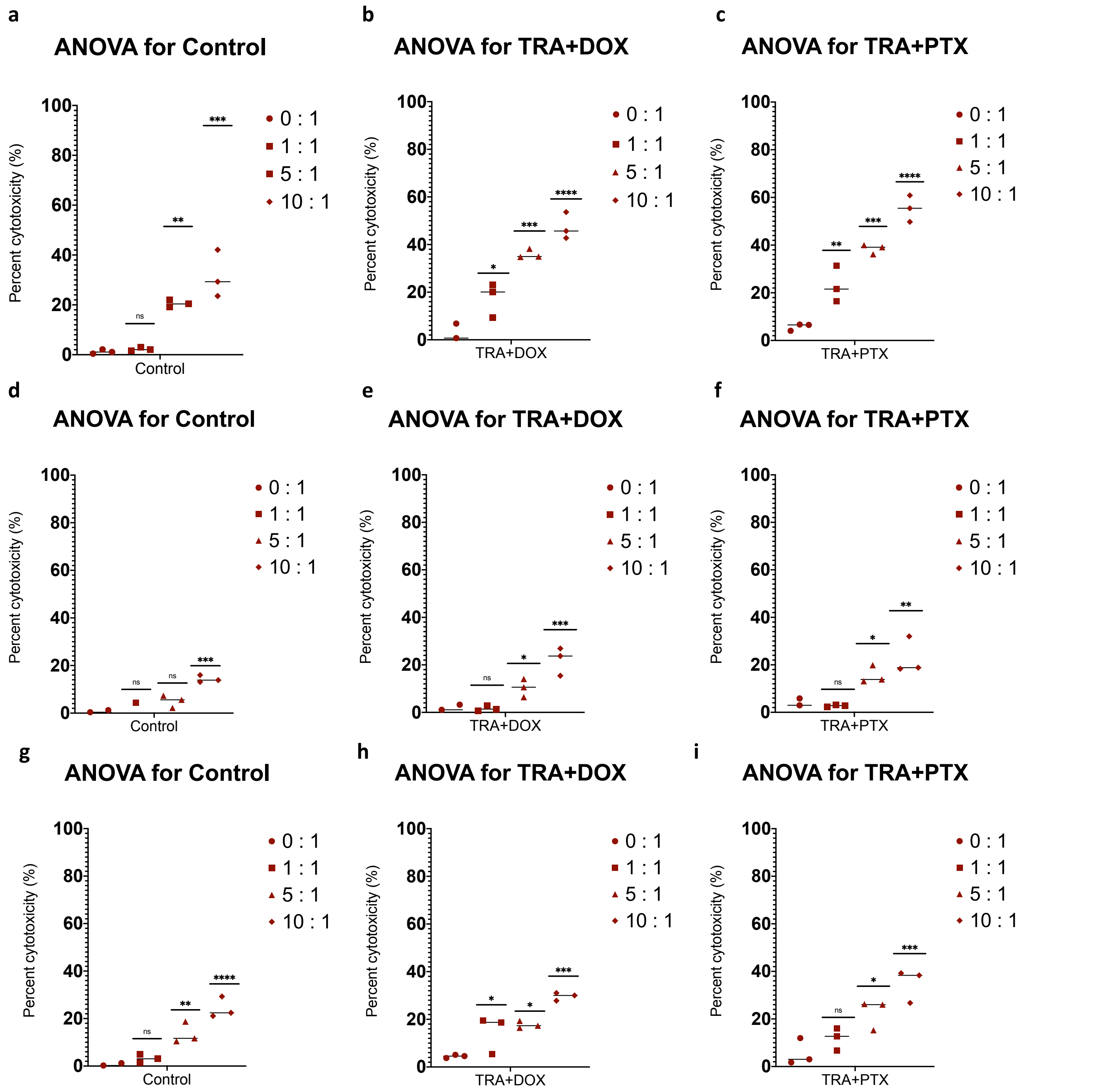

Supplementary Figure 7. ANOVA analysis followed by post hoc Dunnett's test of MiaPaca-2 (a-c), MCF-7 (d-f) and MDA-MB-231 (g-i) spheroids for cytotoxicity experiments. *p ≤ 0.05, **p ≤ 0.01,***p ≤ 0.001, ****p ≤ 0.0001, ns-not significant).

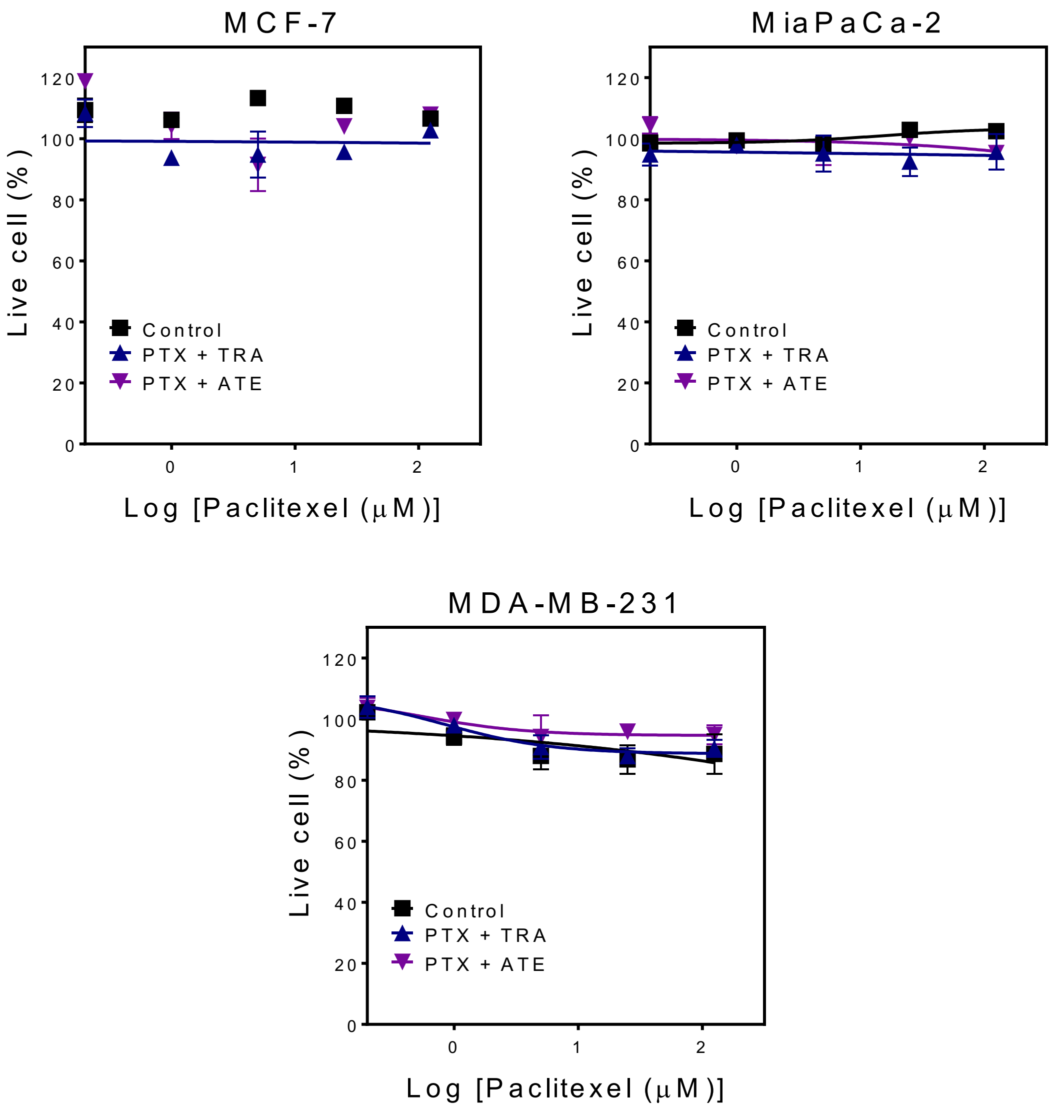

Supplementary Figure 8. Dose-responsive curves for PTX with/without TRA and ATE in the absence of NK-CD16 cells. The error bars represent standard deviation for three replicates.

Supplementary Table 1. EC_{50} values (μM) of PTX for various combination of NK-CD16 cells and antibodies (TRA and ATE).

E : T + Antibody	MCF-7 spheroids	MiaPaCa-2 spheroids	MDA-MB-231 spheroids
0 : 1	> 500	> 500	> 500
0 : 1 + TRA	> 500	> 500	> 500
0 : 1 + ATE	> 500	> 500	> 500
5 : 1	> 500	> 500	> 500
5 : 1 + TRA	272 ± 142[a]	277 ± 114[a]	> 500
5 : 1 + ATE	> 500	12.3 ± 5.8[a]	11.7 ± 7.0[a]

EC_{50} values are obtained from Figure 7b.

[a] Error bars indicate Mean ± SD for three replicates

Supplementary Table 2: Conditions tested for cytotoxicity experiments.

Condition	Effector:Target Ratio	Drugs/Antibody
1	No NK	No Drug/Antibody
2	No NK	2.5 µg/mL of Trastuzumab
3	No NK	3.5 nM Doxorubicin
4	No NK	7.0 nM Paclitaxel
5	No NK	2.5 µg/mL of Trastuzumab+ 3.5 nM Doxorubicin
6	No NK	2.5 µg/mL of Trastuzumab + 7.0 nM Paclitaxel
7	1:1	No Drug/Antibody
8	1:1	2.5 µg/mL of Trastuzumab
9	1:1	3.5 nM Doxorubicin
10	1:1	7.0 nM Paclitaxel
11	1:1	2.5 µg/mL of Trastuzumab + 3.5 nM Doxorubicin
12	1:1	2.5 µg/mL of Trastuzumab + 7.0 nM Paclitaxel
13	5:1	No Drug/Antibody
14	5:1	2.5 µg/mL of Trastuzumab
15	5:1	3.5 nM Doxorubicin
16	5:1	7.0 nM Paclitaxel
17	5:1	2.5 µg/mL of Trastuzumab + 3.5 nM Doxorubicin
18	5:1	2.5 µg/mL of Trastuzumab + 7.0 nM Paclitaxel
19	10:1	No Drug/Antibody
20	10:1	2.5 µg/mL of Trastuzumab
21	10:1	0.0035 µM Doxorubicin
22	10:1	7.0 nM Paclitaxel
23	10:1	2.5 µg/mL of Trastuzumab + 3.5 nM Doxorubicin
24	10:1	2.5 µg/mL of Trastuzumab + 7.0 nM Paclitaxel
25	No NK	Dead Control (Saponin Treated)

Supplementary Table 3: Conditions tested for dose response experiments.

Condition	NK Cells	Paclitaxel Concentration
1	No NK	0 µM
2	No NK	0.02 µM
3	No NK	1 µM
4	No NK	5 µM
5	No NK	25 µM
6	No NK	125 µM
7	5:1	0 µM
8	5:1	0.02 µM
9	5:1	1 µM
10	5:1	5 µM
11	5:1	25 µM
12	5:1	125 µM
13	5:1 + Trastuzumab (2.5 µg/mL)	0 µM
14	5:1 + Trastuzumab (2.5 µg/mL)	0.02 µM
15	5:1 + Trastuzumab (2.5 µg/mL)	1 µM
16	5:1 + Trastuzumab (2.5 µg/mL)	5 µM
17	5:1 + Trastuzumab (2.5 µg/mL)	25 µM
18	5:1 + Trastuzumab (2.5 µg/mL)	125 µM
19	5:1 + Atezolizumab(2.5 µg/mL)	0 µM
20	5:1 + Atezolizumab(2.5 µg/mL)	0.02 µM
21	5:1 + Atezolizumab(2.5 µg/mL)	1 µM
22	5:1 + Atezolizumab(2.5 µg/mL)	5 µM
23	5:1 + Atezolizumab(2.5 µg/mL)	25 µM
24	5:1 + Atezolizumab(2.5 µg/mL)	125 µM
25	No NK	Dead Control (Saponin)
