# Supplementary material for: 3D tumor spheroid microarray for high-throughput, high-content natural killer cell-mediated cytotoxicity
Source: Commun Biol. 2021 Jul 21;4:893. doi: 10.1038/s42003-021-02417-2 (PMC8295284; doi:10.1038/s42003-021-02417-2)
Supplement: Supplementary file 9 — Reporting Summary [file 42003_2021_2417_MOESM9_ESM.pdf]

nature research

Corresponding author(s): Jonathan S. Dordick

Last updated by author(s): Jun 14, 2021

Reporting Summary

Nature Research wishes to improve the reproducibility of the work that we publish. This form provides structure for consistency and transparency in reporting. For further information on Nature Research policies, see our Editorial Policies and the Editorial Policy Checklist.

Statistics

For all statistical analyses, confirm that the following items are present in the figure legend, table legend, main text, or Methods section.

n/a	Confirmed	
☐	☒	The exact sample size (n) for each experimental group/condition, given as a discrete number and unit of measurement
☐	☒	A statement on whether measurements were taken from distinct samples or whether the same sample was measured repeatedly
☐	☒	The statistical test(s) used AND whether they are one- or two-sided<br>*Only common tests should be described solely by name; describe more complex techniques in the Methods section.*
☒	☐	A description of all covariates tested
☒	☐	A description of any assumptions or corrections, such as tests of normality and adjustment for multiple comparisons
☒	☐	A full description of the statistical parameters including central tendency (e.g. means) or other basic estimates (e.g. regression coefficient) AND variation (e.g. standard deviation) or associated estimates of uncertainty (e.g. confidence intervals)
☐	☒	For null hypothesis testing, the test statistic (e.g. F, t, r) with confidence intervals, effect sizes, degrees of freedom and P value noted<br>*Give P values as exact values whenever suitable.*
☒	☐	For Bayesian analysis, information on the choice of priors and Markov chain Monte Carlo settings
☒	☐	For hierarchical and complex designs, identification of the appropriate level for tests and full reporting of outcomes
☒	☐	Estimates of effect sizes (e.g. Cohen's d, Pearson's r), indicating how they were calculated

Our web collection on statistics for biologists contains articles on many of the points above.

Software and code

Policy information about availability of computer code

Data collection	No custom software code used.
Data analysis	No custom software code used.

For manuscripts utilizing custom algorithms or software that are central to the research but not yet described in published literature, software must be made available to editors and reviewers. We strongly encourage code deposition in a community repository (e.g. GitHub). See the Nature Research guidelines for submitting code & software for further information.

Data

Policy information about availability of data

All manuscripts must include a data availability statement. This statement should provide the following information, where applicable:

- Accession codes, unique identifiers, or web links for publicly available datasets
- A list of figures that have associated raw data
- A description of any restrictions on data availability

All data generated or analysed during this study are included in this published article (and its supplementary information files).

Field-specific reporting

Life sciences study design

All studies must disclose on these points even when the disclosure is negative.

Sample size	All experiments were conducted in triplicates.
Data exclusions	Outliers were excluded from analysis. These were primarily any matrigel spots that had detached during the course of the culture.
Replication	Experiments were reproducible across technical replicates.
Randomization	No randomization studies were employed, as they were not relevant for the in vitro work.
Blinding	No blinding studies were employed due to the in vitro study.

Reporting for specific materials, systems and methods

We require information from authors about some types of materials, experimental systems and methods used in many studies. Here, indicate whether each material, system or method listed is relevant to your study. If you are not sure if a list item applies to your research, read the appropriate section before selecting a response.

Materials & experimental systems

n/a	Involved in the study	
☐	☒	Antibodies
☐	☒	Eukaryotic cell lines
☒	☐	Palaeontology and archaeology
☒	☐	Animals and other organisms
☒	☐	Human research participants
☒	☐	Clinical data
☒	☐	Dual use research of concern

Methods

n/a	Involved in the study	
☒	☐	ChIP-seq
☐	☒	Flow cytometry
☒	☐	MRI-based neuroimaging

Antibodies

Antibodies used	ErbB2/Her2 Human anti-Human Alexa Fluor 488, Clone: Hu5, Supplier: R&D Systems, Catalog # FAB9589G-100UG<br>ErbB2/Her2 Human anti-Human, Clone Hu5, Supplier: R&D Systems, Catalog # MAB9589100<br>Human/Mouse/Rat HIF-1 alpha/HIF1A Antibody, Monoclonal Mouse IgG1 Clone # 241809, Catalog # MAB1536<br>Anti-PDL1 antibody, Supplier: VWR, Catalog # 76047-342, Supplier # : A1305-100
Validation	The antibodies are commercially available and have been validated by the company. References are available on the company website under the product.

Eukaryotic cell lines

Policy information about cell lines

Cell line source(s)	MiaPaCa-2, MCF7, MDA-MB-231, NK92-CD16.
Authentication	All the cell lines are commercially available from ATCC.
Mycoplasma contamination	Cell lines are free of Mycoplasma as they were tested.
Commonly misidentified lines (See ICLAC register)	No misidentified lines used.

Flow Cytometry

Plots

Confirm that:

☐ The axis labels state the marker and fluorochrome used (e.g. CD4-FITC).

☒ The axis scales are clearly visible. Include numbers along axes only for bottom left plot of group (a 'group' is an analysis of identical markers).

☐ All plots are contour plots with outliers or pseudocolor plots.

☐ A numerical value for number of cells or percentage (with statistics) is provided.

Methodology

Sample preparation	The cells were washed with DPBS and treated with FITC conjugated antibody in 1% BSA in DPBS. The cells were washed 3X times and resuspended in DPBS. The cell sample was then run on flow cytometer
Instrument	BD-LSRII
Software	BD FACS DIVA<br>FlowJo
Cell population abundance	Cell populations were from individual cell types and not a mixture in our flow cytometry. Each cell type was run separately to provide 100% purity.
Gating strategy	Gates were generated by running cells without any antibody. The FSC/SSC and FITC gates were drawn based on this negative control population

☒ Tick this box to confirm that a figure exemplifying the gating strategy is provided in the Supplementary Information.
