# Supplementary material for: 3D tumor spheroid microarray for high-throughput, high-content natural killer cell-mediated cytotoxicity
Source: Commun Biol. 2021 Jul 21;4:893. doi: 10.1038/s42003-021-02417-2 (PMC8295284; doi:10.1038/s42003-021-02417-2)
Supplement: Supplementary file 10 — Description of Supplementary Files [file 42003_2021_2417_MOESM10_ESM.pdf]

Description of Additional Supplementary Files

File Name: Supplementary Data 1

Description: Data for Figure 1d.

File Name: Supplementary Data 2

Description: Data for Figure 2c.

File Name: Supplementary Data 3

Description: Data for Supplementary Figure 3a and b.

File Name: Supplementary Data 4

Description: Data for Figure 6b and c

File Name: Supplementary Data 5

Description: Data for Supplementary Figure 7a-i.

File Name: Supplementary Data 6

Description: Data for Figure 7a and b.

File Name: Supplementary Data 7

Description: Data for Supplementary Figure 8 and Supplementary Table 1.
